# Concurrent mapping of brain ontogeny and phylogeny within a common space: Standardized tractography and applications

**DOI:** 10.1126/sciadv.abq2022

**Published:** 2022-10-19

**Authors:** Shaun Warrington, Elinor Thompson, Matteo Bastiani, Jessica Dubois, Luke Baxter, Rebeccah Slater, Saad Jbabdi, Rogier B. Mars, Stamatios N. Sotiropoulos

**Affiliations:** ^1^ Sir Peter Mansfield Imaging Centre, School of Medicine, University of Nottingham, Nottingham, UK.; ^2^ Centre for Medical Image Computing, Department of Computer Science, University College London, London, UK.; ^3^ Université Paris Cité, Inserm, NeuroDiderot Unit, Paris, France.; ^4^ University Paris-Saclay, CEA, NeuroSpin, Gif-sur-Yvette, France.; ^5^ Department of Paediatrics, University of Oxford, Oxford, UK.; ^6^ Wellcome Centre for Integrative Neuroimaging, University of Oxford, Oxford, UK.; ^7^ Donders Institute for Brain, Cognition, and Behaviour, Radboud University, Nijmegen, Netherlands.; ^8^ National Institute for Health Research (NIHR) Nottingham Biomedical Research Centre, Queens Medical Centre, Nottingham, UK.

## Abstract

Developmental and evolutionary effects on brain organization are complex, yet linked, as evidenced by the correspondence in cortical area expansion across these vastly different time scales. However, it is still not possible to study concurrently the ontogeny and phylogeny of cortical areal connections, which is arguably more relevant to brain function than allometric measurements. Here, we propose a novel framework that allows the integration of structural connectivity maps from humans (adults and neonates) and nonhuman primates (macaques) onto a common space. We use white matter bundles to anchor the common space and use the uniqueness of cortical connection patterns to these bundles to probe area specialization. This enabled us to quantitatively study divergences and similarities in connectivity over evolutionary and developmental scales, to reveal brain maturation trajectories, including the effect of premature birth, and to translate cortical atlases between diverse brains. Our findings open new avenues for an integrative approach to imaging neuroanatomy.

## Teaser

An integrative brain mapping framework reveals linked and unique connectivity changes due to development and evolution.

## INTRODUCTION

Developmental and evolutionary effects on the brain and its organization occur at vastly different time scales, yet these effects have been shown to be linked (*1*, *2*). For instance, allometric changes in cortical area expansion show notable correspondence across ontogeny and phylogeny. Brain regions that expand later in newborn humans are also those that differ the most in size between humans and monkeys (*2*). However, it is still not possible to study concurrently the ontogeny and phylogeny of cortical areal connections, which is arguably more relevant to brain function than allometric measurements (*3*). Mapping these changes across multiple domains and diverse brains is inherently challenging.

Magnetic resonance imaging (MRI) provides unique capabilities for noninvasive brain mapping, applicable to both the human and nonhuman brain, and across the life span. Traditional methods have approached the problem of comparison of diverse brains and their organization as a geometrical alignment task, by attempting image registration of, for example, cortical folding landmarks (*4*). There is a number of shortcomings in this approach. First, sulci that are often used to align adult brains together are largely absent in nonhuman primates and less developed in (preterm) neonates. Second, alignment based on geometrical landmarks alone does not ensure functional correspondence, even within the same species and age group (*5*). For instance, well-studied regions such as the primary visual cortex can vary up to twofold in areal size across individuals (*6*), and this functional variability cannot be captured by cortical folding alone.

Alternative methods have recently been introduced (*7*, *8*), including contributions from our group (*8*, *9*), which use brain connections to proxy similarities and differences in brain organization across species. Regions that have similar functional specialization are anticipated to have similar patterns of extrinsic (i.e., interregion) connections (*3*, *10*). By comparing the pattern of structural or functional connections of brain areas, estimated using diffusion MRI (dMRI) or resting-state functional MRI, respectively, it becomes possible to compare brains in a latent “connectivity space” that is not dependent on the geometry of different brains (*10*).

We have previously demonstrated that one can describe each part of brain’s cortical gray matter in terms of its unique pattern of extrinsic connections to a set of landmarks, provided by white matter fiber bundles (*8*, *11*). Major fascicles can be reliably identified through dMRI tractography in diverse brains, such as in humans and macaques (*12*). Importantly, however, the projections of these bundles to gray matter (i.e., the cortex) will differ. The patterns of how gray matter locations connect to these bundles can be compared across brains and can be used to probe brains’ phylogeny.

**Fig. 1.**
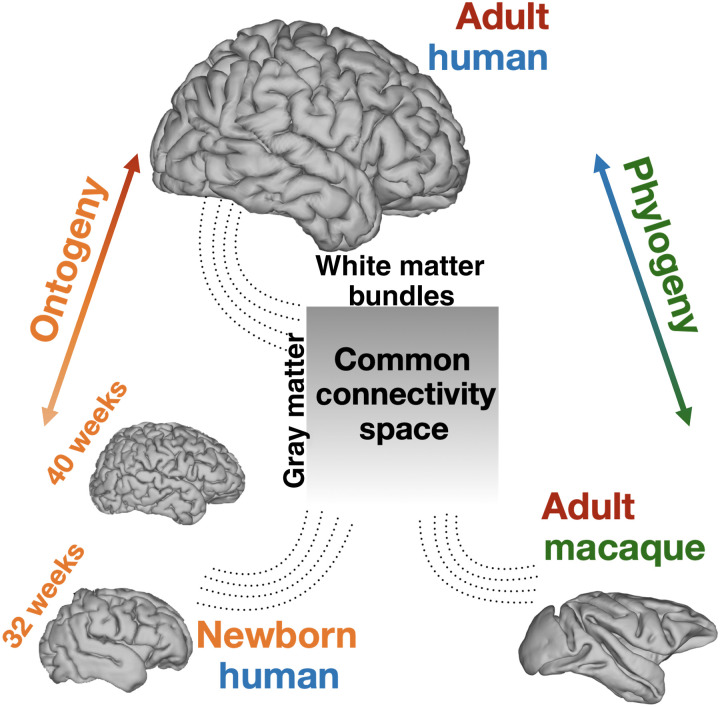
Mapping diverse brains into a common connectivity space using white matter fiber bundles as “landmarks.” This allows for definitions of cortical gray matter connectivity patterns with respect to the white matter fiber bundles and comparisons across both ontogeny and phylogeny. We use diffusion MRI data and devise tractography protocols for delineating corresponding white matter bundles across neonatal humans, adult humans, and macaques to define this common connectivity space.

In this study, we build upon these ideas and tackle the challenge of integration across both phylogeny and ontogeny of brain connections. We present a framework that allows us to concurrently map brain connectivity from humans (adults and neonates) and nonhuman primates (macaque monkeys) onto a common space, anchored on white matter bundles. Toward this, we define and construct a new library of tractography protocols for mapping 42 white matter bundles in neonates from dMRI data (baby-XTRACT), ensuring correspondence to protocols previously defined for the adult human and macaque brains (XTRACT) (*12*). We use the uniqueness of cortical areal connection patterns to these bundles to probe areal specialization (Fig. 1), and we demonstrate the feasibility of concurrently mapping three diverse brains onto this common space.

This framework enables us to quantitatively study divergences and similarities in cortical connectivity over both evolutionary and developmental scales. In the context of evolutionary developmental biology (evo-devo), we investigate whether regions whose connections are developed later in humans coincide with regions whose connection patterns differ most greatly between humans and monkeys. We investigate changes in connectivity with development by comparing the brains of neonatal (across different gestational ages) and adult humans and explore whether the development of brain connectivity might be modulated by extrinsic/environmental factors such as premature birth. Last, we demonstrate how we can use our framework to translate cortical atlases between diverse brains. Together, these contributions open new and exciting possibilities for untangling the brain’s complexity in standardized ways that have not been possible before.

## RESULTS

### Neonatal tract protocols and atlases

**Fig. 2.**
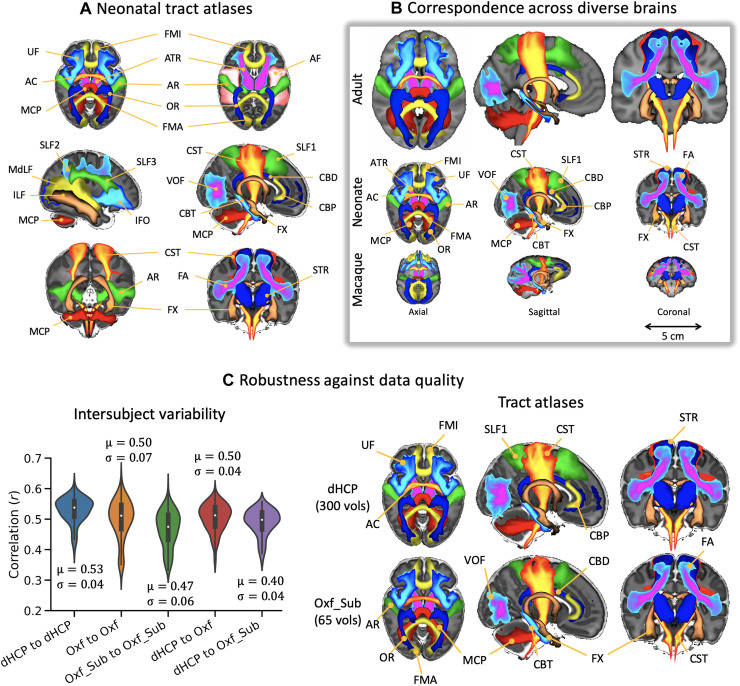
Neonatal white matter tract reconstruction, correspondence with adult human and macaque tracts, and robustness across independent diffusion MRI datasets. (**A**) Axial, sagittal, and coronal views of population percentage atlases of 42 tracts from 277 full-term dHCP neonates. The tract atlases were created by averaging binarized (at a threshold of 0.1%) path density maps across subjects, obtained from probabilistic tractography. For ease of visualization, all tracts are displayed as maximum intensity projections with 30 to 100% population coverage. Tract names and abbreviations are provided in Table 1. (**B**) Tract atlases from the adult human, neonatal human, and macaque brain. Visualization is the same as in (A). (**C**) Left: Intersubject variability of tract delineations across three neonatal independent datasets: dHCP (300 diffusion volumes, 1.5-mm isotropic resolution), Oxford (163 diffusion volumes, 1.75-mm isotropic resolution), and Oxford-subsampled (65 diffusion volumes, 1.75-mm isotropic resolution). Each violin plot is a distribution of 231 correlations between pairs of subjects, averaged across all tracts, within and across datasets. Right: Neonatal tract atlases from a subset of 22 age- and sex-matched subjects from independent datasets [dHCP (top row) versus Oxford-subsampled (bottom row)].

We developed a novel library of standardized tractography protocols for 42 major white matter bundles of the neonatal brain (which we call baby-XTRACT), including commissural, association, projection, and limbic tracts (Table 1 in Materials and Methods). Crucially, these protocols were defined in consistency with previous protocols (XTRACT) (*12*) for the same bundles in the adult human and macaque, using similar gray and white matter definitions for bundle delineation. Figure 2A shows neonatal tract atlases obtained by applying the protocols to high-quality dMRI data of 277 full-term newborns [scanned at 37 to 45 weeks postmenstrual age (PMA)] from the Developing Human Connectome Project (dHCP) (*13*). Looking into narrower age ranges (full terms scanned at 37 to 40, 40 to 42, and 42 to 45 weeks PMA), we could confirm that the tractography protocols produced highly consistent results across these groups (fig. S1). Figure 2B shows qualitatively how the neonatal tract delineations compare against the ones defined before in the adult brain and in the nonhuman primate brain.

**Table 1.** The 42 tracts included in XTRACT. Description of the 42 tracts included in XTRACT, along with their abbreviations used to refer to them in the text. Bilateral tracts have separate protocols for their left and right counterparts. The reverse seeding approach is used for some tracts, whereby the protocols are run twice with the seed and target masks reversed. Reverse seeding is used in all cases for the macaque brain but not in all cases for the adult/neonate brains.

**Category**	**Tract name**	**Abbreviation**	**Bilateral (yes/no)**	**Reverse seeding (yes/no)**
**Association fibers**	Arcuate fasciculus	AF	Yes	Yes
	Frontal aslant tract	FA	Yes	Yes (macaque only)
	Inferior fronto-occipital fasciculus	IFO	Yes	Yes
	Inferior longitudinal fasciculus	ILF	Yes	Yes
	Middle longitudinal fasciculus	MdLF	Yes	Yes
	Superior longitudinal fasciculus 1, 2, and 3	SLF1, SLF2, and SLF3	Yes	Yes (macaque only)
	Uncinate fasciculus	UF	Yes	Yes (macaque only)
	Vertical occipital fasciculus	VOF	Yes	Yes
**Commissural fibers**	Anterior commissure	AC		Yes
	Forceps major (splenium of the corpus callosum)	FMA		Yes
	Forceps minor (genu of the corpus callosum)	FMI		Yes
	Middle cerebellar peduncle	MCP		Yes
**Limbic fibers**	Cingulum bundle: dorsal section	CBD	Yes	Yes (macaque only)
	Cingulum bundle: perigenual section	CBP	Yes	Yes (macaque only)
	Cingulum bundle: temporal section	CBT	Yes	Yes (macaque only)
	Fornix	FX	Yes	Yes (macaque only)
**Projection fibers**	Acoustic radiation	AR	Yes	Yes
	Anterior thalamic radiation	ATR	Yes	Yes (macaque only)
	Corticospinal tract	CST	Yes	Yes (macaque only)
	Optic radiation	OR	Yes	Yes
	Superior thalamic radiation	STR	Yes	Yes (macaque only)

We tested the applicability and robustness of these tractography protocols using independent neonatal dMRI data from a different scanner and with different acquisition parameters. In addition to the dHCP dataset (high angular and spatial resolution, bespoke setup, and subset of neonates scanned at 37 to 42 weeks PMA), we used two further datasets (from neonates also scanned at 37 to 42 weeks PMA): (i) a dataset from a conventional clinical scanner (no specialized acquisition hardware/software, lower angular and spatial resolution than dHCP— “Oxford dataset”) and (ii) a subsampled version of the Oxford dataset [keeping only *b*≤1000 s/mm^2^ to mimic a standard diffusion tensor imaging (DTI) acquisition—“Oxford-subsampled dataset”]. For acquisition parameters and data quality, see table S1, and for cohort details, see fig. S9. We compared the tract atlases and the intersubject variability across these three datasets (after age and sex matching). Strong similarity was observed between the average tract atlases, shown in Fig. 2C (right and fig. S2), allowing good neonatal tract delineations even with a standard data acquisition protocol. Some differences can be observed between the datasets, for instance, reduced tract extent in the second branch of the superior longitudinal fasciculus (SLF2) and lower population coverage in the acoustic radiation (AR) in the Oxford-subsampled dataset. However, the average spatial correlation across all tracts in the group atlases was 0.89 (SD, 0.04) between the dHCP and Oxford datasets and 0.86 (SD, 0.08) between the dHCP and Oxford-subsampled datasets.

Intersubject variability in the tractography results was assessed within and across the subject groups (relative to the dHCP dataset). As shown in Fig. 2C (left), intersubject variability was relatively consistent across the datasets with, as expected, greater intersubject similarity within than across groups, albeit with more variance in the Oxford and Oxford-subsampled datasets. For the remaining analyses, we used the dHCP data, due to its better quality (table S1) and larger cohort.

**Fig. 3.**
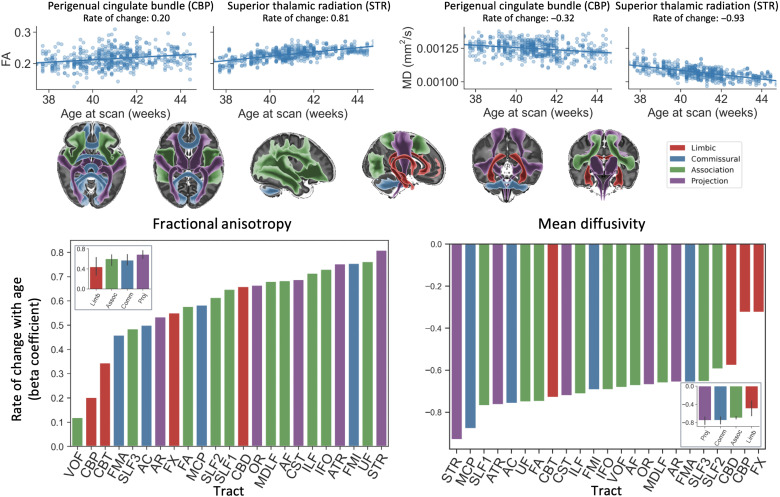
White matter tract maturation. Top: Linear regression of tract-averaged microstructural (DTI) metrics (left, FA; right, MD) with neonatal age (37 to 45 weeks PMA) across 277 full-term neonates. Examples are shown for fast changing [superior thalamic radiation (STR)] and a slow changing [perigenual cingulum bundle (CBP)] tract. Bottom: The rate of change with age for the given microstructural measure (left, FA; right, MD), estimated as the beta coefficients from a general linear model between tract-wise microstructure and gestational age, and the mean (and SD) beta values for each tract type (inset bar plots). Tracts are color-coded by tract type, and the mean regression beta coefficients are provided for each tract type. Birth weight, head circumference, tract volume, and QC score have been used as confounds in the regressions.

We further investigated whether the developed neonatal tractography protocols could capture early developmental trends in tract maturation. Projection fibers (e.g., thalamocortical and corticothalamic) are expected to mature more quickly over this early life period, followed by commissural fibers (e.g., corpus callosum), association fibers (e.g., superior longitudinal fasciculi), and limbic fibers (e.g., cingulum) (*14*–*18*). We mapped tract-averaged microstructure parameters [mean diffusivity (MD) and fractional anisotropy (FA)] with neonatal age (37 to 45 weeks PMA at scan) using general linear models. A significant increase in FA and reduction in MD with age was observed for all tracts (Fig. 3). The rate of change with age for the given microstructure measure (Fig. 3, bottom) reflected the asynchronous maturation of different tracts. Although some mixing between broad categories was observed, projection tracts showed more rapid changes, whereas limbic bundles changed more slowly over this period, with commissural and association tracts having intermediate rates of change. These trends agree, in general, with expectation. Together, the above analyses demonstrate that the neonatal tractography protocols give reproducible tracts across independent datasets and stages of development and can capture known neurodevelopmental trends.

### Extracting connectivity patterns and mapping divergence across ages and species

Correspondence across diverse brains (adult humans, neonate humans, and macaques) is a key feature in our tractography protocol definitions. We used these corresponding tracts as landmarks to define a common connectivity space, within which we could perform brain comparisons. All of the considered tracts exist in the different brains, given their early development in humans and their stability across phylogeny/among primates, but the way cortical gray matter connects to these white matter tracts varies. Hence, we not only have common features to use as a reference (the tracts) but also have differences to compare (pattern of connectivity to these tracts).

We used connectivity blueprints (*8*) to enable these comparisons (see Materials and Methods for full details). These are (cortex × tracts) matrices that represent how different gray matter locations are connected to a set of white matter tracts. Using our tractography protocols, we constructed these connectivity maps, anchored on the 42 corresponding tracts provided by our tractography protocols for adult humans, neonate humans, and macaques.

**Fig. 4.**
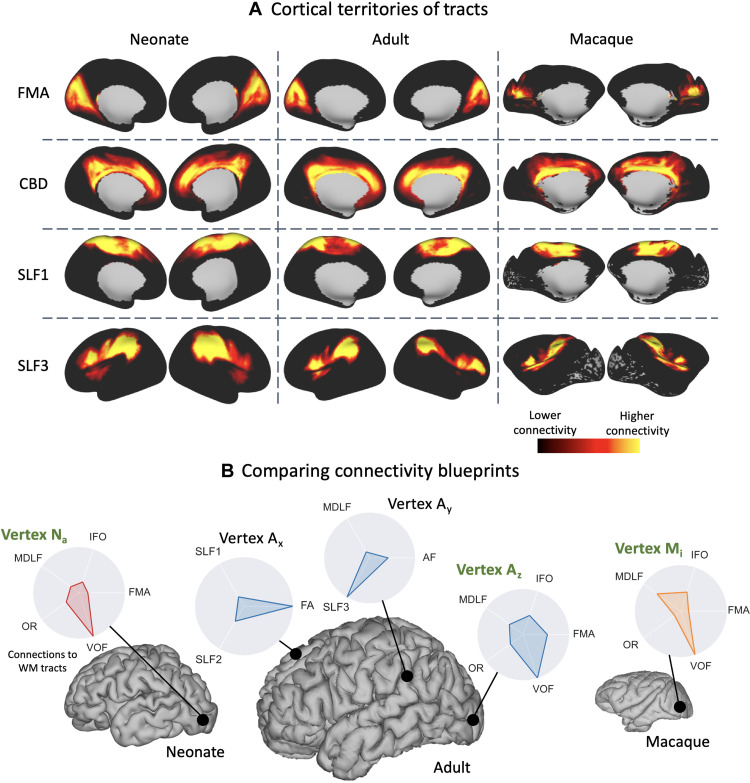
Building a common connectivity space across the neonatal human, adult human, and macaque brain using patterns of cortical connections to corresponding white matter tracts. (**A**) Examples of the cortical territories of white matter tracts derived for the neonate human (averaged across 33 neonates born and scanned at 40 weeks PMA), adult human (50 random young adult HCP subjects) and macaque brain (6 high-quality postmortem brains) (not to scale). These maps correspond to columns of the connectivity blueprints (see Materials and Methods and Fig. 8). (**B**) The patterns of connections of different cortical gray matter locations to white matter tracts may be compared across diverse brains, even in the absence of geometrical correspondence, using measures of statistical similarity. These patterns correspond to rows of the connectivity blueprints. In the presented example, the best-matching pattern to vertex N_a_ in the neonatal brain is identified in the adult human (A_z_) and macaque (M_i_) brains by sweeping through all vertices in the adult/macaque brain, resulting in a pair of cortical locations in the occipital region with strong VOF projections.

A column of the connectivity blueprint represents the cortical territories of a tract, with examples across different brains shown in Fig. 4A. Consequently, a row of the connectivity blueprint describes the pattern of how a given cortical location connects to the set of considered tracts (Fig. 4B). Given the built-in correspondence of the tracts, normalized connectivity patterns may be treated as probability distributions in the same “sample space.” They can then be compared across diverse brains using measures of statistical similarity, e.g., the symmetric Kullback-Leibler (KL) divergence (*19*). Using this definition, we expect that regions with similar connectivity patterns to these tracts to appear close in this common connectivity space. Because the pattern of connections is linked to the functional role of a brain region (*3*), this space can therefore probe functional similarity and divergence. An example is shown in Fig. 4B where the matching vertex to a location in the neonatal occipital cortex N_a_ is found in the adult human and macaque brains by sweeping through the connectivity patterns of all vertices in the adult/macaque brains and identifying the one (A_z_ for the adult human and M_i_ for the macaque, both in the occipital cortex) with most similar pattern to N_a_ (i.e., by finding the vertex with the minimum KL divergence). All vertices predominantly connect to the vertical occipital fasciculus (VOF), with connections also to the optic radiation (OR), inferior fronto-occipital fasciculus (IFO), middle longitudinal fasciculus (MDLF), and corpus callosum (FMA); while, for instance, vertex A_x_ in the adult human superior frontal cortex connects more to association tracts.

**Fig. 5.**
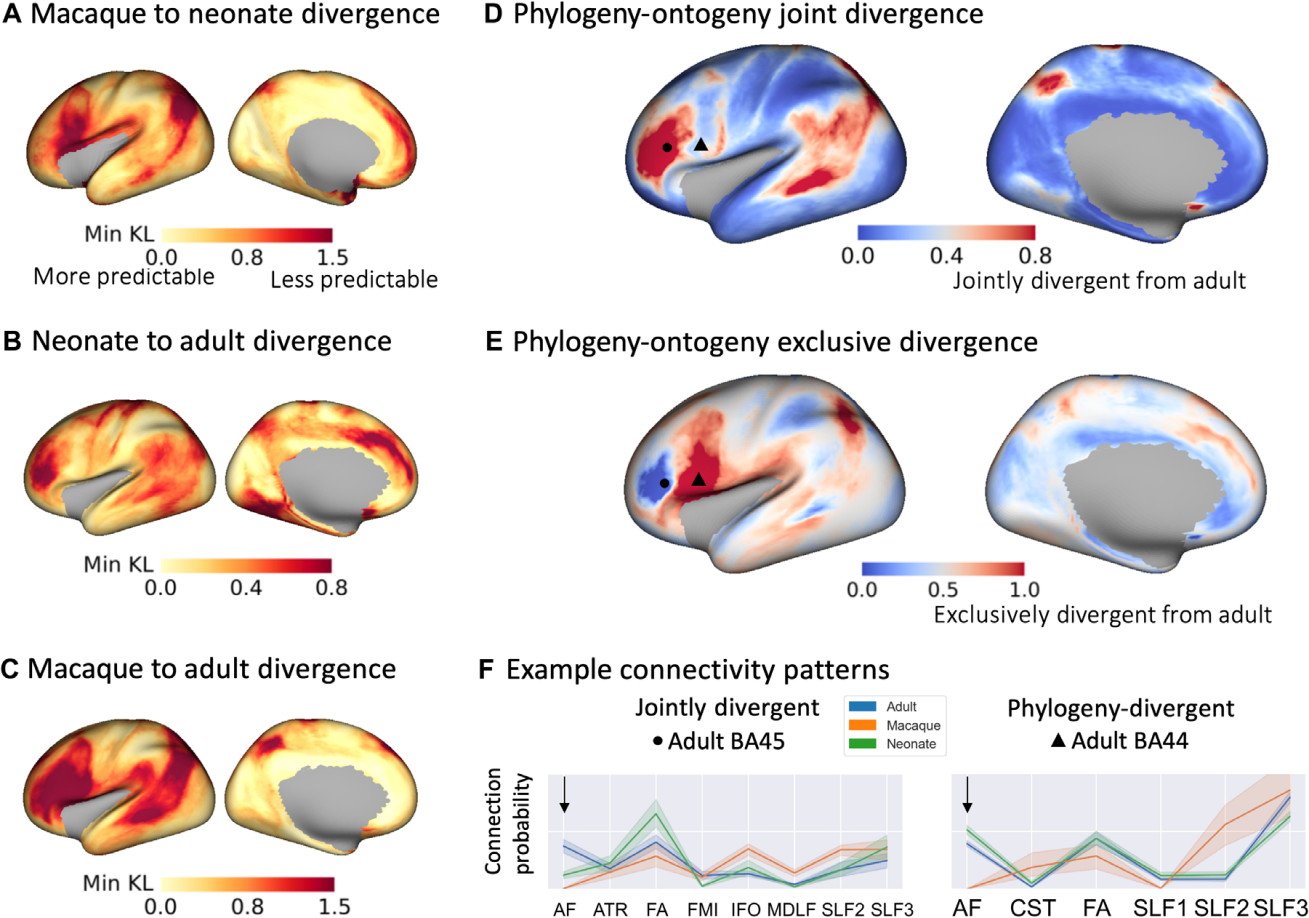
Divergence of connectivity patterns between human macaque (phylogeny) and human adult neonate (ontogeny) not only share similar patterns but also exhibit unique features. (**A** to **C**) Divergence (minimum KL divergence) was calculated for each vertex, comparing between the above groups, i.e., across the ontogeny (B) and phylogeny (C) dimension. Group blueprints were used (33 neonates born and scanned at 40 weeks PMA, 50 adult HCP subjects, and 6 macaque animals). Small divergence values correspond to regions with more predictable connectivity patterns between the two considered groups. (**D**) Phylogeny-ontogeny joint divergence map, calculated as the product of (B) and (C) (B × C) with larger (red) values indicating that divergence to the adult is greater both across phylogeny and ontogeny. This indicates regions that develop later in life and have evolved in primates. (**E**) Phylogeny-ontogeny exclusive disjoint (exclusive OR) divergence map, calculated as the (B + C) − 2(B × C) (union minus the intersection). Larger (red) values indicate regions that either develop later in humans or have evolved in primates, but not both. (**F**) Connectivity patterns for example vertices in the inferior frontal region (Brodmann areas 44 and 45): A vertex in the region of interest (ROI) was selected on the adult human surface [marked with a dot and a triangle respectively in (D) and (E)], the corresponding minimum KL divergence vertex on the neonate and macaque surface was identified, and the connectivity pattern was plotted for each example and each brain. The line represents the mean connectivity pattern across subjects for each group, and the shaded area is the 95% confidence interval. In Brodmann area 45 (left plot), patterns were jointly divergent (i.e., for both neonates and macaques) from adult humans. In Brodmann area 44 (right plot), patterns were more divergent for macaques, while for neonates and adult humans, they are quite similar. The arrow highlights these differences for connections through the arcuate fasciculus (AF). For visualization, only the most highly contributing tracts are displayed (tract contribution of >0.05 to any group).

We performed comparisons across both the ontogeny and phylogeny dimensions using this common connectivity space. Connectivity blueprints were constructed for groups of adult [50 random Human Connectome Project (HCP) subjects], macaque (six high-quality postmortem macaque datasets), and neonate brains, and KL divergence was used to assess similarities and divergences. To avoid confounding our analysis with the effects of neonatal development, incidental findings or prematurity, we used a subset of full-term neonates (33 neonates, born and scanned at 40 weeks PMA, with no incidental findings). Figure 5 (A to C) shows the minimum KL divergence maps for pairs of groups. By finding the minimum KL divergence for each cortical location, i.e., by asking how different is the best matching connectivity profile of a given area across brains, we could assess predictability in connectivity patterns between groups.

We found higher divergence when comparing across species [mean minimum KL divergence of 0.62 (SD 0.38) and 0.68 (SD 0.44) between the macaque and neonate and macaque and adult, respectively; Fig. 5, A and C] rather than within species [mean minimum KL divergence of 0.33 (SD 0.19) between neonate and adult human; Fig. 5B]. Between the neonate and adult human, the highest divergence was observed in the inferior and medial frontal, temporal, and inferior parietal regions. These regions reflect maximum dissimilarity in connectivity patterns of newborns compared to later in life, therefore indicating regions that are less developed/matured at birth and develop later. Comparing the human and macaque brain, the inferior frontal, temporal, and parietal regions were mostly divergent, reflecting regions that have evolved across primate species. To assess the robustness of these findings, we performed a bootstrap analysis, repeatedly calculating the minimum KL divergence between randomly subsampled groups. We found a similar pattern in divergence (fig. S3) between all tested pairs, although with greater variance in phylogeny maps, perhaps due to the greater divergence observed in these comparisons or due to the limited macaque sample size.

To explore whether areas with high connectivity divergence are the ones with high connectivity complexity, we explored the association between divergence maps and entropy in connectivity blueprints (i.e., complexity of connection patterns of cortical regions) (fig. S4). We found some association between divergence and entropy maps, with some strong local correlations. However, it was not the case that high divergence was fully driven by high complexity of connection patterns.

We subsequently mapped how the divergence of connectivity patterns compare jointly across ontogeny and phylogeny. Figure 5D shows a joint divergence map of the neonate and macaque with respect to the adult human [i.e., product of maps in Fig. 5 (B and C), corresponding roughly to the union of the two sets]. High values in this map correspond to regions whose connections develop/mature later in humans and also emerged later (more recently) in human evolutionary history. Figure 5E shows an exclusive disjunction (exclusive OR) map of the two patterns, highlighting where one of the ontogeny and phylogeny divergences are high with respect to the other but not both of them. The similarity pattern shown in Fig. 5D is impressively close to previous results based on cortical expansion in human development and between human and nonhuman primates (*2*). Frontal, parietal, and temporal regions that have evolved in humans from primates tend to mature more slowly. Figure 5F provides example connectivity patterns for selected vertices in the inferior frontal region where sharp gradients in ontogeny-phylogeny divergence are observed. On the left, patterns are jointly divergent, i.e., in both neonates and macaques, they diverge from the corresponding pattern in the adult humans. On the right, patterns are divergent between macaques and humans, while for neonate and adult humans, they are quite similar. We see that the arcuate fasciculus (AF), a fascicle involved in the production and understanding of language, is a key driving factor in these maps, in line with expectations from the literature on evolution (*20*, *21*) and development (*22*–*24*).

### Exploring developmental changes with respect to the adult brain

**Fig. 6.**
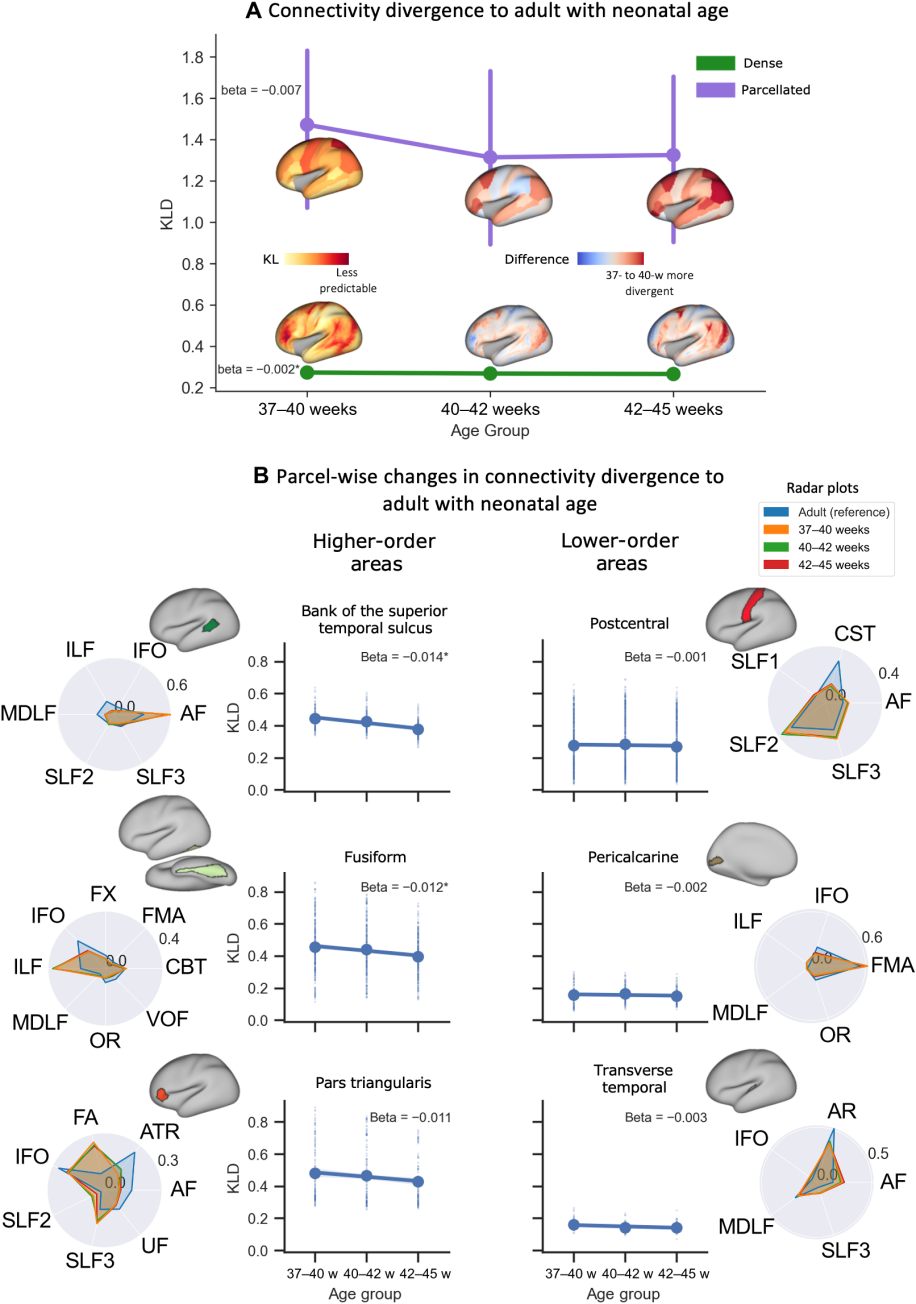
Divergence between neonatal and adult brain connectivity patterns decreases on average with development but exhibits regionally variable rate of change. (**A**) Divergence was calculated for three neonatal age groups (37 to 40, 40 to 42, and 42 to 45 weeks PMA, 73 subjects in each group) relative to the adult brain. The whole-brain median (and median absolute deviation) is plotted against neonatal age at two spatial levels: (i) the dense level (bottom surface plots), finding the minimum KL divergence between any two vertices; and (ii) the parcellated level (top surface plots), where the dense KL divergence matrix was parcellated using the Desikan-Killiany (DK) cortical atlas, and the divergence between corresponding parcels is found. Surface plots represent the KL divergence map for the first age group and, subsequently, the difference between the first age group and each other age group. Red values indicate greater divergence in the 37- to 40-week neonate compared to the age of interest; blue indicates reduced divergence. (**B**) Changes in divergence of connectivity patterns between adults and neonates against neonatal age, for example, regions: higher-order associative (left column) and lower-order sensory (right column) regions (distributions extracted from dense divergence maps, each dot is a vertex). Radar plots show the parcel-averaged tract connectivity to those parcels for each neonatal age group and the adult brain (see figs. S5 and S6 for all regions). For visualization, only the most highly contributing tracts are displayed (tract contribution of >0.05 to any group), and each plot area has been sum-normalized. Beta values correspond to the rate of change in KL divergence with neonate age derived via linear regression. The parcel mean is indicated by the large dot. Asterisk indicates significant trends after Bonferroni correction for multiple comparisons.

A key feature in our common space framework is the ability to compare brains with respect to a reference. We investigated connectivity changes at different neonatal ages with respect to the adult brain, shown in Fig. 6. This allowed us to tackle the challenging task of using a fully mature brain as a reference for different early developmental stages.

We constructed group-averaged connectivity blueprints for the neonatal brain at three different stages of early development (37 to 40 weeks, 40 to 42 weeks, and 42 to 45 weeks PMA at scan, again excluding those with incidental findings and any premature subjects). Figure 6A shows the average divergence for these different developmental neonatal stages against age, for both dense (vertex-wise) and parcellated (region-wise) reconstructions. For parcellated comparisons, we applied the Desikan-Killiany (DK) cortical atlas to the KL divergence matrices for both the adult and neonate and compared corresponding parcels. On average, a decrease in divergence, relative to the adult brain, was observed with increasing age, as indicated by the regions/locations shown in red in the difference maps for older age groups in Fig. 6A. Even if the level of divergence reduction exhibited regional variations, it was evident throughout the brain, revealing the development and maturation of cortical connections even in this relatively brief period. At a dense level (i.e., vertex-wise), the overall changes were small on average, but they were enhanced with (reduced spatial scale) parcel-wise comparisons.

Changes in the divergence of connectivity patterns between the adult and neonatal brain against neonatal age are highlighted in Fig. 6B, for example, regions (all regions shown in figs. S5 and S6). The rate of change with age exhibits regional variability, but an interesting pattern emerges from these examples. On the left column, a set of higher-order areas (associative multimodal regions such as the superior temporal sulcus, fusiform area, and pars triangularis) show greater overall divergence relative to the adult and more rapid changes with age. These trends are indicative of more rapid development of connectivity during this early life period. On the right, cortical regions lower in cortical hierarchy (primary unimodal regions such as sensorimotor, visual, and auditory) display overall lower divergence relative to the adult and slow changes with age. These trends can be indicative of greater maturity in the connections of these regions.

### Probing differences due to preterm birth within the common connectivity space

To study the vital scientific question of how premature birth affects brain connectivity, we used our approach to explore differences between the full-term and very preterm (age at birth <32 weeks PMA) brain, scanned at full term–equivalent age. Premature birth is a major burden worldwide and is well known to lead to significant disruptions in neurodevelopment throughout life (*25*). The common connectivity space enables unique explorations into how preterm and full-term neonates differ, using the adult brain as a reference for full maturation.

**Fig. 7.**
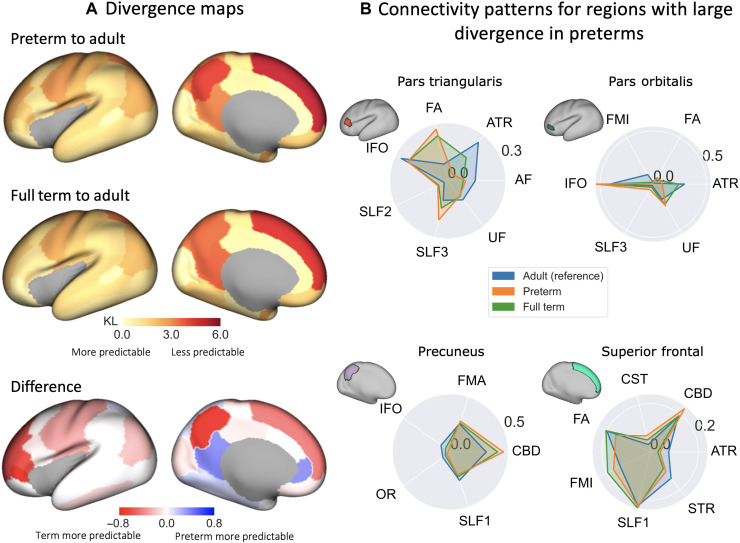
Connectivity patterns of premature neonates are more dissimilar than full-term neonates, relative to the adult brain. (**A**) KL divergence maps and their between-group difference. KL divergence matrices were calculated between the preterm and adult (top) and full-term and adult (middle) group connectivity blueprints (25 age- and sex-matched neonates per group). Divergence matrices were then parcellated using the DK cortical atlas and the divergence between corresponding parcels found. The difference between the preterm and full-term divergence maps (bottom; i.e., full term–preterm), with red indicating greater divergence in the preterm brain compared to the full-term brain, relative to the adult brain. (**B**) Tract connectivity profiles [adult (blue), preterm (orange), and full term (green)] for a subset of parcels of interest with large between-group differences. For visualization, only the most highly contributing tracts are displayed (tract contribution of >0.05 to any group), and each plot area has been sum-normalized.

Group-averaged connectivity blueprints were calculated for the two subgroups of neonates (25 in each group, age- and sex-matched—full details in fig. S9). The neonatal connectivity blueprints were then compared through KL divergence to the adult connectivity blueprint, and the KL divergence between corresponding DK parcels was calculated (Fig. 7A). Higher divergence was observed between the adult and preterm brain (mean of 1.74) than between the adult and the full-term brain (mean of 1.63). This suggests that the connectivity patterns in the preterm brain (scanned at term-equivalent age) are, on average, less similar to those of the adult brain, compared to the full-term brain.

The superior frontal, inferior frontal (pars triangularis and orbitalis), and precuneus areas showed maximum divergence differences to the adult brain, suggesting that these areas are more dissimilar between preterms and adults than between full terms and adults. The connectivity profiles for these regions are presented as polar plots in Fig. 7B. Large differences in frontal connectivity were driven to a large extent by reduced connectivity in preterms to the anterior thalamic radiation (ATR) and AF. This agrees with previous findings that frontal white matter “quality” (maturation and development) is reduced in the preterm infant (*18*, *26*, *27*). We also observed differences both in the sensorimotor cortical regions (results not shown) and superior frontal regions, driven in part by differences in the superior thalamic radiation (STR). This agrees with previous work that thalamic connections are less developed in the preterm infant (*28*). Differences in the precuneus were mostly driven by underrepresentation of association tracts in preterms (e.g., SLF1 and IFO) and corresponding overrepresentation of the cingulum bundle. Some regions in the limbic system (parahippocampal and rostral and isthmus cingulate parcels) showed the opposite trend, where preterms demonstrated connectivity patterns more similar to the adult compared to full term to adult comparison.

### Connectivity embedding for cross-species, cross-ages atlas translation

Connectivity blueprints further allow for a direct translation of cortical atlases between geometrically diverse brains (*8*). Using the similarity (or inverse KL divergence) of connectivity patterns as a metric, a low-dimensional embedding can be achieved. Regions with similar connection profiles will appear close to each other, with dimensions of the embedding representing maximum variability in similarity patterns. Therefore, likely equivalent areas are expected to group together, even if their location and size vary across brains.

**Fig. 8.**
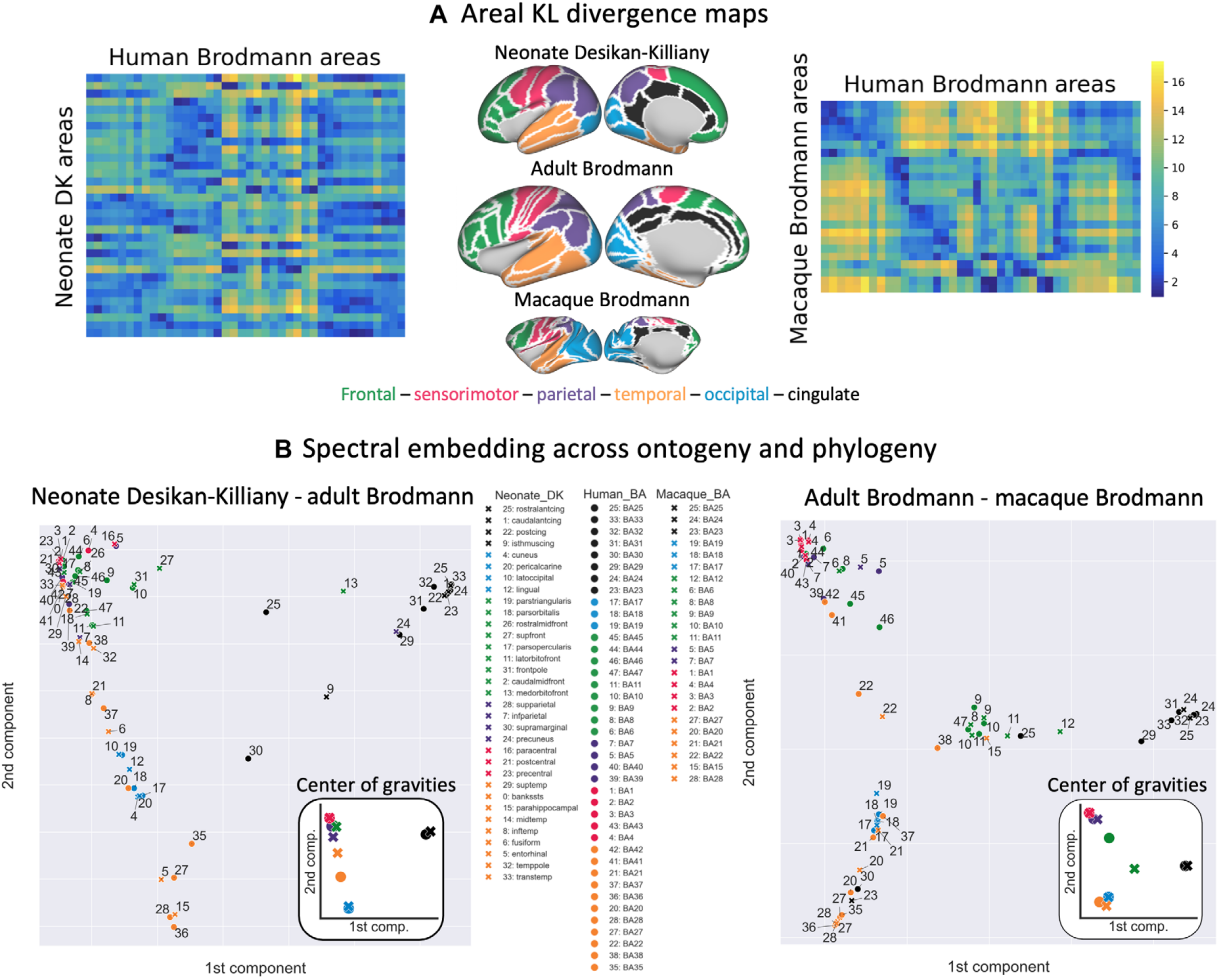
Translating cortical atlases across species and ages using spectral embedding of connectivity. (**A**) KL divergence matrices were calculated between group (neonate and adult, adult, and macaque) connectivity blueprints. Divergence matrices were then parcellated using the Brodmann cortical atlas for the adult and macaque brain and the DK cortical atlas for the neonatal brain, taking the median value for each region. For visualization, we further define a set of anatomofunctional cortical systems, colored-coded in the middle column surface plots. (**B**) The inverse of the KL divergence matrices (i.e., the similarity) was then used as a feature set in spectral embedding and the first two components projected into a 2D space, color-coded by major brain regions, for the neonate-adult (left) and macaque-adult (right) embeddings. Circles represent the Brodmann parcels for the adult brain, and crosses represent the DK parcels for the neonate brain (left) and the Brodmann parcels for the macaque brain (right). Figure insets show the center of gravity (median of parcel coordinates) for each anatomofunctionally defined cortical system for each brain. cing, cingulate; front, frontal; temp, temporal; trans, transverse; med, medial; mid, middle; inf, inferior; lat, lateral; ant, anterior; post, posterior. For visualization, the embedding plots are reproduced in a larger format in the Supplementary Materials (figs. S7 and S8).

We used such an embedding to identify phylogeny and ontogeny correspondences between cortical atlases of the neonatal and macaque brain with the surface format Brodmann parcellation of the adult brain (*29*). For the macaque brain, we used the surface format Brodmann vervet monkey atlas (*29*). For the neonatal brain, we used the DK atlas (*30*). Through spectral reordering (*31*), the inverse of the KL divergence matrices of pairs of blueprints (Fig. 8A) (neonate to adult and macaque to adult) were projected to low-dimensional spaces. We used the top two modes of variation to define a two-dimensional (2D) space (Fig. 8B, reproduced in a larger format in figs. S7 and S8), within which each region can be represented with the component weights of its connectivity profile.

Overall, parcels from similar anatomofunctional cortical systems (color-coded in Fig. 8A) tended to group together, both across species and across ages. Sensorimotor and occipital regions did so more, while frontal regions were more scattered. For instance, adult primary visual Brodmann areas 17, 18, and 19 showed the smallest distance to neonatal primary visual regions (pericalcarine, cuneus, and lateral occipital in DK parcellation) and to monkey visual Brodmann areas 17, 18, and 19 and the greatest distance to areas that do not receive any visual projections, such as the cingulate areas (for neonates—rostral, caudal, anterior, and isthmus cingulate in DK parcellation; for monkeys—cingulate areas 24 and 25) and sensorimotor areas (for neonates—paracentral, postcentral, and precentral in DK parcellation, and for monkeys—sensorimotor Brodmann areas 1 to 4). We took the center of gravity in the embedded connectivity space for each color-coded cortical system and for each brain (Fig. 8B, insets). The occipital (blue) and sensorimotor (pink) regions were closest for both the neonate-adult and macaque-adult embedded spaces, reflecting that the lower-order cortical areas show close similarity in their connections across ontogeny and phylogeny compared to other regions. The largest distance was observed between the adult human and macaque frontal regions, driven in part by the inferior frontal parcels (Brodmann areas 44 to 46), as expected given their apparent uniqueness to the human brain. Together, these results demonstrate how different atlases from brains across species and across ages can be related to each other using the common connectivity space.

## DISCUSSION

A significant hurdle toward a fully integrative approach to neuroanatomy is the lack of unifying frameworks that would allow comparisons between diverse brains, correspondence between brain atlases, and compatible terminology between different subfields. This markedly impedes translational investigations aiming to bridge developmental and comparative neuroscience (*32*–*34*). Here, we tackled this challenge by proposing a novel framework, based on standardized tractography protocols, which integrates connectivity maps from humans (adults and neonates) and nonhuman primates (macaques) and enables quantitative comparisons in cortical connectivity over both evolutionary and developmental scales. Key to this framework is the idea of describing nonhuman primate, human adult, and human newborn brains all in a single common space (*35*), consisting of homologous white matter fiber bundles that can be unambiguously identified in all of them, even if their cortical terminations differ.

To achieve this, we first developed and tested a novel library of tractography protocols for reconstructing white matter bundles in the neonate brain (baby-XTRACT). Previous studies have developed neonatal protocols (*14*, *36*–*38*); however, none have been developed where correspondence across diverse brains is explicit. The protocols developed here are defined analogously with protocols for the adult human and macaque brain (*12*). We demonstrated that these protocols are reliable for developmental data, generalizable across a range of acquisition parameters and data qualities, and we are making them openly available to the community. We also used the resultant white matter bundles to explore tract maturation in neonates and found trends that generally agree with expectations from the literature (*14*–*18*).

Subsequently, the in-built correspondence in the white matter bundle delineation protocols was exploited to anchor a common connectivity space and to provide a means for performing direct comparisons across ontogeny and phylogeny. Although the old notion that ontogeny is a full “replay” of evolution has now been discredited, it seems that areas of the cortex that have expanded most in human lineage (multimodal associative regions as opposed to primary unimodal regions) are the ones that tend to develop/mature latest in development (*39*, *40*). Consistent with previous observations based on cortical expansion (*2*) and microstructural maturation (*41*), we showed that there are a number of cortical territories in the frontal, temporal, and parietal regions whose connections both develop later in the human brain and have a different pattern in the human brain compared to the macaque (and, crucially, these territories are not simply the ones with higher connectivity complexity, as shown by the entropy maps—fig. S4). These included regions that have previously been suggested to be particularly well developed in the human, including the anterior prefrontal cortex (*42*, *43*) and inferior parietal lobule (*44*). Crucially, and previously undescribed, we also found cortical territories where the two dimensions of ontogeny and phylogeny do not converge. An interesting case is provided by the left hemisphere inferior frontal region hosting Broca’s area. This region is recognized to consist of distinct subdivisions (*45*), with different cytoarchitecture, transmitter receptor distribution, and connectivity. Here, we showed that the posterior and anterior part of Broca’s area seem to have distinct connections in the human brain compared to the macaque, but it is the anterior part that seems to show a later maturation of connections. These results have implications for the development of higher cognitive abilities in humans, as caudal parts of this larger territory are commonly ascribed a role in phonological/motoric aspects of language production, while more rostral parts are thought to have more semantic/lexical roles, although the specificity of functional localization is debatable (*46*).

Our approach allowed us to use the divergence in connectivity patterns between the neonate and adult brains to explore changes linked to early development at different gestational stages (37 to 45 weeks PMA) (Fig. 6). Assessing neonatal development this way allows us to contextualize changes during this early stage of development with respect to the fully mature adult brain. We found that cortical regions lower in cortical hierarchy (sensorimotor, visual, and auditory) have already more mature connectivity, whereas connectivity for higher-order regions (frontal, parietal, and temporal regions) develops more rapidly during this period. Note that maturation of connectivity patterns presented here (i.e., of how cortical areas connect to different white matter bundles during early development) does not necessarily follow the microstructural maturation trend of specific tracts presented in Fig. 3 (i.e., how dense/myelinated each tract is during early development), providing complementary views. For instance, the microstructure of projection tracts matures with higher rates, yet the preferential connectivity pattern of these tracts to sensorimotor areas seems to have already been developed and mature more slowly over this developmental period, perhaps reflecting differences in axonal growth rates, white matter maturation, and cortical development (e.g., folding) (*47*). Mapping developmental changes, as demonstrated here for the human brain, can be extended to nonhumans, for example, as it has been recently done for the macaque brain (*48*), thus augmenting the dimensionality of our framework to capture developmental trajectories for multiple species. Hence, although we restrict our analysis to the development of the neonate in this paper, our common space approach may be used to explore development across the life span of the human and other species.

We further tested the effect of premature birth on brain connectivity between full-term and preterm neonates, scanned at full-term equivalent age. We found higher divergence in the preterm brain compared to full-term brain, relative to the adult brain. These differences are generally larger for the superior and inferior frontal, medial, and inferior parietal regions and sensorimotor regions and agree with previous studies (*18*, *26*–*28*), yet our framework allows for direct comparisons against the adult brain. These divergence maps seem to follow overall the trends observed in Fig. 6, with the preterm divergence map appearing to represent a further underdeveloped neonatal brain; the mean whole-brain KL divergence in the preterm brain is greater than the 37- to 40-week neonate, and the same pattern of divergence is observed. However, we also find apparent increases in connectivity divergence in the full-term brain compared to the preterm brain in the parahippocampal and rostral and isthmus cingulate parcels. It is unclear whether these findings are a true reflection of anatomical differences between the groups. These may reflect the acceleration of maturation due to stressors (i.e., premature exposure to the extrauterine environment) (*49*–*51*), although this would contradict the reported disruptions to limbic development in preterm newborns and in association with early neonatal stress (*25*, *52*). These apparent accelerations may also occur because of limitations in image alignment in the presence of anatomical abnormalities, e.g., enlarged ventricles, which are more common in our preterm subgroup. Alternatively, these findings may be linked to limitations of the automated neonatal DK parcellation. For regions bordering the medial wall (and particularly the isthmus cingulate and parahippocampal regions), we observed that the automatically identified parcels and their boundaries looked overly inclusive in neonates compared to expectation. Therefore, results for these regions need to be interpreted with caution.

We demonstrated another powerful application of the common connectivity space in using it to achieve a low-dimensional connectivity embedding within which atlases across different brains could be translated. Specifically, we used connection patterns to link cortical parcellation atlases of the neonatal, adult human, and macaque brains that lacked a built-in a priori correspondence. We found that parcels from similar cortical systems clustered together, regardless of the parcellation scheme used. For instance, visual areas across brains and cortical atlases clustered together in the low-dimensional embedded space and were separate from, e.g., cingulate areas that do not receive visual tract projections. These translations may be extended to any map of cortical features reflecting organization and hierarchy at different levels (*8*, *9*), such as cortical myelination maps or even maps of functional activation where correspondence in activation patterns is expected (*53*).

In using white matter tracts as landmarks for the common connectivity space, we alleviated known issues in tractography, particularly in estimating end-to-end (i.e., gray matter to gray matter) connections (*54*, *55*). First, we used anatomical priors to define protocols for well-documented white matter bundles, focusing on the body of the tracts. These are much clearer to identify reliably (*56*, *57*) and can be identified across species and ages. Second, having established the bodies of the tract, we used a novel procedure to estimate the gray matter projections of the tracts. The most obvious approach of tracking toward the gray matter has the problem that one moves through bottlenecks in the cortical gyrus and after which fibers fan out. Most tractography algorithms have problems resolving this fanning, leading to what is known as the gyral bias (*58*). However, we took the opposite approach of tracking from the gray matter surface toward the white matter, thus following the direction in which the fibers are expected to merge, rather than to fan out. We then multiplied the surface–to–white matter tractogram with that of the body of the tract to create the connectivity blueprint. This avoids some of the major problems associated with tracking toward the surface (*8*).

A limitation of the current study is the use of a prespecified set of white matter fiber bundles to describe cortical connectivity patterns. Even if these protocols sample representative groups of white matter fibers and provide relatively high cortical coverage to contrast the effects of phylogeny and ontogeny, they do not fully represent connectivity patterns from all cortical areas. For instance, the insular cortex was excluded from our analyses. Further work could either define more standardized protocols for the extraction of additional fiber bundles or use data-driven approaches for the extraction of denser connectivity components (*11*). Another limitation is that we did not replicate the ontogeny/phylogeny analyses using the independent neonate datasets (Oxford and Oxford-subsampled). We expect, however, that the observed trends will hold given high correlation between neonatal cohort atlases. Last, in using the baby-XTRACT protocols to obtain tract maturation trends, we only captured a snapshot of the rate of maturation through a given stage of development. We did not consider the maturation stage (i.e., amount of maturation already taken place) for each tract, which is expected to vary across tracts, according to the brain regions connected. For instance, among projection fibers, the corticospinal tract (CST) and OR are expected to show earlier maturation than fibers to and from the frontal lobe, while limbic tracts have already undergone intense maturation by this age (*15*, *41*).

A number of interesting future explorations, bridging developmental and comparative neuroscience questions, arise from this study. Here, we explored ontogeny in humans only and within a relatively brief window of neonatal development, up to 45 weeks PMA. Future work could not only expand this to explore the full developmental trajectory from neonates, through childhood and adolescence, and to older adults, as aging does not influence all brain systems equally (*59*); but also expand to study ontogeny for some nonhuman primates and certain nonprimate mammals. Tractography protocols, similar to those presented in our study, have been developed for additional nonhuman primates (*60*, *61*) and the pig brain (*62*), providing the seed for such future studies.

Furthermore, the present method of describing cortical gray matter in terms of their pattern of connectivity with the major white matter bundles of the brain can also be used to compare lateralization across the cortical hemispheres in phylogeny/ontogeny comparisons. In (*12*), we showed differences in the connectivity pattern between hemispheres of the adult human brain in frontal and temporoparietal areas reached by the arcuate fascicle and superior longitudinal fascicles. This dovetails with results showing differences in tract volumes, FA, and stream counts of these fiber bundles in adults and children (*56*, *63*). However, it remains to be established whether our approach and these traditional measures correlate across subjects. Moreover, the presence of cortical lateralization in nonhuman primates is controversial (*20*, *64*, *65*) and requires a larger sample size of macaques than used in the present investigation.

Last, exploring how individual variability in connectivity patterns across human brains interacts with the trends observed here would be an interesting future direction. In our study, we mostly kept explorations at the group level as a means to “deconfound” the ontogeny and phylogeny results from individual differences (but see Fig. 5 and fig. S3). However, it has been argued that individual variability is found in the same places as variation across primate species (*64*), presumably because evolution exploits the variation across individuals. Studying individual differences in disease can also be envisaged. For instance, it has been hypothesized that “evolutionary” bundles (i.e., those that are common across species but show evolutionary change) are particularly vulnerable to diseases that are uniquely human, such as schizophrenia (*66*, *67*).

In summary, we have (i) developed and validated protocols for the neonatal brain (baby-XTRACT), standardized against the adult human and macaque, and release these openly; (ii) demonstrated feasibility of using these new protocols and the ones published previously by our group to concurrently map the brains of three diverse groups into a common space; (iii) concurrently mapped divergence of connectivity patterns across ontogeny and phylogeny; (iv) shown how very different cortical atlases for neonates, adult humans, and macaques can be linked with spectral embedding; (v) explored neonatal development with respect to the mature adult brain; and (vi) explored the effects of prematurity, using the adult brain as a fully matured reference. Our common space framework allows us to explicitly test hypotheses across multiple dimensions of brain connectivity in an integrative manner and identify connectivity patterns that are linked to developmental and evolutionary differences.

## MATERIALS AND METHODS

### Neonatal tractography protocols (baby-XTRACT)

Tractography protocols for neonates were defined following the general principles of XTRACT (*12*), ensuring direct correspondence with the adult human and the macaque brain. In total, 42 white matter tracts (19 bilateral and 4 commissural) were defined for the neonatal brain (Table 1).

Protocols consisted of a set of rules and regions of interest (ROIs), drawn in standard space, which govern tracking. These included seed (streamline starting points), target/waypoint (region through which a streamline should pass to be valid), exclusion (regions that reject any streamline passing through them), and stop/termination (regions that stop tracking if a streamline passes through them) masks. During tractography, these standard space protocol masks are warped to native space where tractography is performed, and the subsequent paths are resampled back to standard space during tracking, in a way that minimizes resampling.

To ensure correspondence with the XTRACT protocols, the MNI-space adult protocols were used as a very initial starting point. A nonlinear warp field was used to roughly align the adult protocol masks to a 40-week PMA neonatal template (*68*). These registered masks were then manually redrawn to ensure good alignment and correspondence to the neonatal anatomy. To avoid artificial lateralization in bilateral tracts, seed and target masks were enforced to have equal volumes in each hemisphere. Additional revisions were made to the protocols based on preliminary results, to optimize the results for the neonatal anatomy. The full protocol descriptions are provided in Supplementary Text.

In some protocols, we implemented a reverse-seeding approach. Here, the protocol was run twice, with the roles of the seed and target masks exchanged. The resultant streamline distributions were then added together. We chose to go with a “single-seeding” strategy for some protocols, only when the reverse and single-seeding approaches converged, as single-seeding requires about half the computational resources (see Table 1).

### Data and preprocessing

#### Neonatal data

dMRI data were drawn from 438 neonates born at 24 to 43 and scanned at 29 to 45 weeks PMA, made publicly available by the second data release of the dHCP (*13*). Briefly, data were acquired during natural sleep on a 3T Philips Achieva with a dedicated neonatal imaging system, including a neonatal 32-channel head coil (*13*). dMRI data were acquired over a spherically optimized set of directions on three shells (*b* = 400, 1000, and 2600 s/mm^2^). A total of 300 volumes were acquired per subject, including 20 with *b* = 0 s/mm^2^, across four acquisition subsets (two pairs with opposing phase encoding polarities). For each volume, 64 interleaved overlapping slices were acquired (in-plane resolution = 1.5 mm, thickness = 3 mm, and overlap = 1.5 mm). The data were then superresolved (*69*) along the slice direction to achieve isotropic resolution of 1.5 mm^3^ and preprocessed to correct for motion and distortions (*70*, *71*). The distortion-corrected dMRI data were separately linearly aligned to the T2-weighted space and the concatenation of the diffusion-to-T2 and T2–to–age-matched template transforms allowed diffusion–to–age-matched template warp fields to be obtained (*72*). The dHCP data release includes an assessment of incidental findings scored 1 to 5 (the “radiology score”) with larger values indicating larger or more clinically significant incidental findings. For some analyses, we used this scoring system to exclude subjects with a score of >3 (3 indicates “incidental findings with unlikely clinical significance but possible analysis significance”). For a full description of the subgroups used in each analysis, see fig. S9.

#### Adult data

We drew from the preprocessed, publicly released HCP dMRI data (*73*). We randomly chose 50 unrelated subjects (age range, 22 to 35 years). Briefly, the HCP data were acquired using a bespoke 3T Connectom Skyra (Siemens, Erlangen) with a monopolar diffusion-weighted (Stejskal-Tanner) spin-echo echo planar imaging (EPI) sequence, an isotropic spatial resolution of 1.25 mm, three shells (*b* values = 1000, 2000, and 3000 s/mm^2^), and 90 unique diffusion directions per shell plus 6 *b* = 0 s/mm^2^ volumes, acquired twice with opposing phase encoding polarities. Protocol details are summarized in table S2. Nonlinear transformations to the MNI152 standard space were obtained using T1-weighted images with FSL’s FNIRT (*74*). The distortion-corrected dMRI data were separately linearly aligned to the T1-weighted space, and the concatenation of the diffusion-to-T1 and T1-to-MNI transforms allowed diffusion-to-MNI warp fields to be obtained.

#### Macaque data

We used six high-quality postmortem macaque (age range, 4 to 16 years) datasets in this study, as described previously (*12*). These data were acquired at 7T using a Agilent DirectDrive console (Agilent Technologies, Santa Clara, CA, USA) using a 2D diffusion-weighted spin-echo protocol with single-line readout, with 16 *b* = 0 volume, 128 volumes acquired with *b* = 4000 s/mm^2^, and an isotropic spatial resolution of 0.6 mm. Protocol details are summarized in table S2. Nonlinear transformations to the macaque standard space (F99) (*75*) were estimated using FSL’s FNIRT (*74*) based on the FA maps.

#### Fiber orientation estimation and tractography

Fiber orientations were modeled for up to three orientations per voxel using FSL’s BEDPOSTX (*76*) and used to inform tractography. For the neonatal brain, a model-based deconvolution against a zeppelin response kernel was used to accommodate for the low anisotropy inherent in data from this age group (*71*).

Probabilistic tractography was performed using FSL’s XTRACT (*12*), which uses FSL’s PROBTRACKX (*77*), with streamlines seeded from and constrained by the protocol masks, as described in the “Tractography (baby-XTRACT) protocol definitions” section (in Supplementary Text). A curvature threshold of 80° was used, the maximum number of streamline steps was 2000, and subsidiary fibers were considered above a volume fraction threshold of 1%. A step size of 0.5 mm was used for the neonatal and adult brain, and a step size of 0.2 mm was used for the macaque brain. Resultant path distributions were normalized by the total number of valid streamlines.

### Generation of population white matter tract atlases

Tractography results from groups of subjects were used to obtain tract atlases, in the form of population percentage overlap. The normalized path distributions for each tract were binarized and then averaged across subjects. The resultant spatial maps describe the percentage of subjects for which a given tract is present at a given voxel. We generate our main high-quality tract atlases using all 277 full-term and “normally appearing” (i.e., no analysis-significant incidental findings) dHCP neonates. In addition, to explore robustness of our protocols across developmental stages, we generate atlases for three different neonatal age groups (37 to 40, 40 to 42, and 42 to 45 weeks PMA at scan) with 73 neonates in each group. For full group details, see fig. S9.

### Robustness against neonatal data quality

To explore the robustness of the protocols across data of varying quality and acquisition parameters, we use an additional dataset of 22 neonates born and scanned at 37 to 42 weeks PMA (full details in fig. S9) (*78*), taking the dHCP dataset as the benchmark dataset. These data were collected on a 3T Siemens Prisma with a nonspecialized adult 32-channel receive coil. dMRI data were acquired with 163 volumes per subject, over three shells (*b* = 500, 1000, and 2000 s/mm^2^), with 1.75-mm isotropic voxels and an acquisition time of 8 min. This dataset was acquired at the Wellcome Centre for Integrative Neuroimaging (Oxford, UK) and is referred to as the Oxford dataset.

As a further test, a third dataset was generated by removing the *b* = 2000 s/mm^2^ shell from the Oxford dataset and reducing the overall number of volumes to 65 (which corresponds to an approximate scan duration of 3 min). This corresponds to a more conventional low–*b* value acquisition and will be referred to as the Oxford-subsampled dataset. The acquisition parameters of the three datasets are summarized in table S1.

These datasets were analyzed following the dHCP data preprocessing as described above, including motion and distortion correction, the generation of diffusion-template warp fields, crossing-fiber modeling, and standardized tractography for each subject. We compared tract-atlases and intersubject variability across the three datasets. A group of dHCP neonates, matched for age and sex, was selected for comparison. Tract atlases were compared quantitatively by correlating each tract (with population threshold of 30% applied) from the dHCP dataset with the respective tracts from the comparison datasets. Intersubject variability in the tractography results was assessed within and across the subject groups. Similarity was assessed using the Pearson’s correlation coefficient between subjects’ normalized tractography maps in template space, thresholded at 0.1%. The correlation values were averaged across tracts for each subject pair. For within-group comparisons, the subjects were each compared with each of the other subjects in the group, yielding 231 pairs of subjects. For across-group comparisons, 231 pairs were randomly generated across the groups to give the same number of data points.

### Microstructure of white matter tracts in early development

We used the developed library of neonatal tractography protocols to explore early developmental trends of white matter microstructure, specifically, the rate of maturation at birth. We investigated changes in tract-averaged DTI parameters (FA and MD) with age.

To reduce confounds caused by, e.g., prematurity, we restricted our subject pool to a group of 277 full-term infants from the dHCP dataset, born at 37 to 42 weeks, and scanned at 37 to 45 weeks PMA (see fig. S9 for full details). The diffusion tensor model was fitted to the *b* = 1000 s/mm^2^ shell for each subject, and the voxel-wise FA and MD maps were calculated. The tract atlases derived from the dHCP dataset were thresholded at 30% and binarized. These tract masks were then warped to each subjects’ native diffusion space and used as ROIs to calculate tract-wise median FA and MD values.

A general linear model was then used to assess the relationship between tract-wise microstructure (FA and MD) and the subjects’ gestational age at scan in weeks, including birth weight (in grams), head circumference at scan (in centimeters), tract volume in native space (in cubic millimeters—sum of nonzero voxels following registration of thresholded atlas to native space), and data quality control (QC) score as confounding regressors. The QC score was the *z*-score average of the signal-to-noise ratio and contrast-to-noise ratio of the subject’s dMRI data (*13*).

### Building connectivity blueprints

**Fig. 9.**
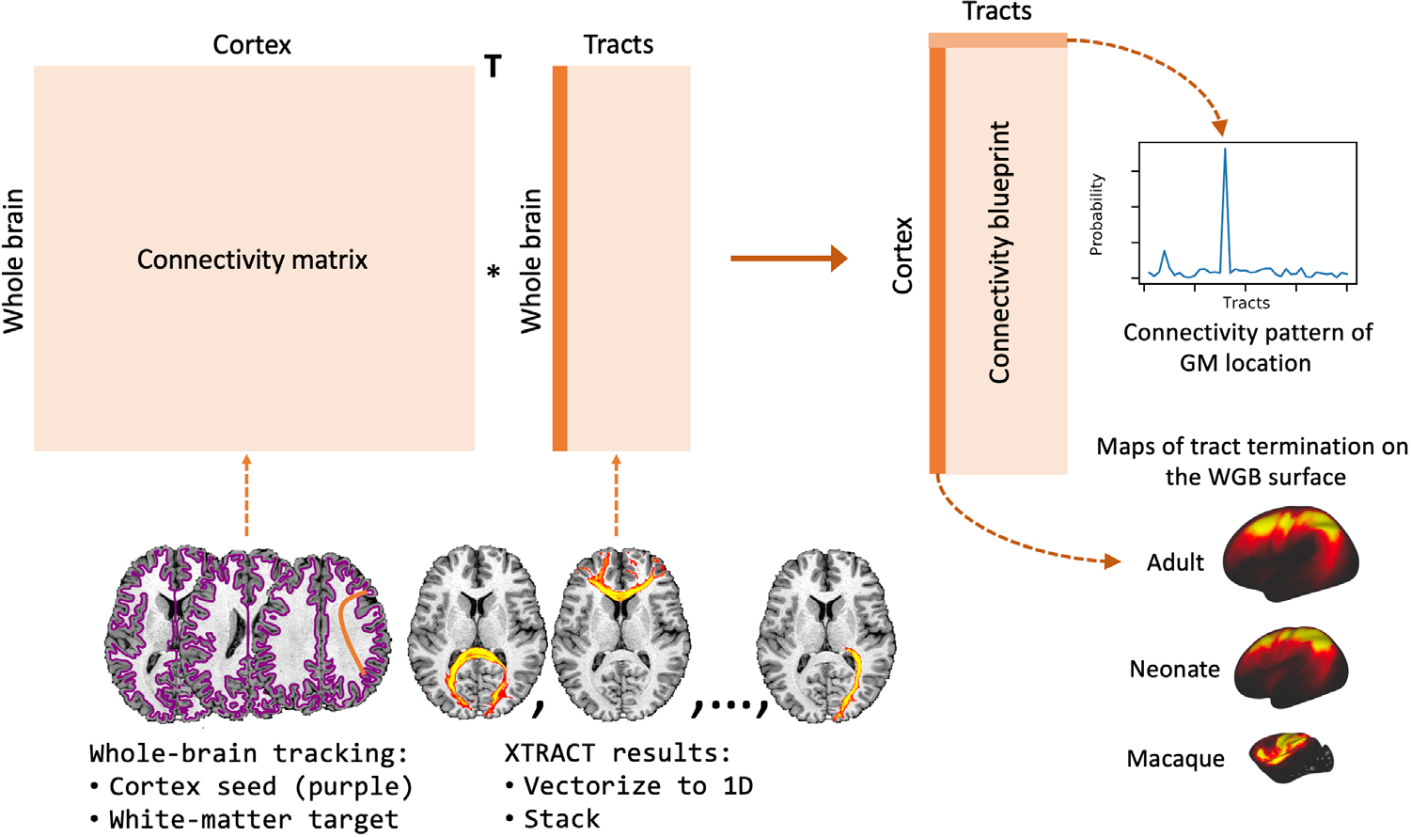
Building connectivity blueprints. Connectivity blueprints (right) are calculated by taking the dot product of a cortex–to–whole brain white matter connectivity matrix (left) with a matrix of tractography maps, unwrapped to 1D (middle). Columns of the connectivity blueprint provide maps of the cortical territories of tracts and rows consist of cortical connectivity patterns, describing how each cortical location is connected to the white matter tracts.

Tractography results can be used to generate maps of the cortical termination of each tract, using connectivity blueprints (*8*). The process of extracting these connectivity patterns is shown in Fig. 9. Tractography results are unwrapped to 1D, yielding a (whole brain × tracts) matrix. Next, whole-brain probabilistic tractography is performed to build a (cortex × whole brain) connectivity matrix, seeding streamlines from the cortical white matter–gray matter boundary (WGB) and counting visitations to each white matter voxel. Connectivity blueprints (cortex × tracts) are derived as the product of this whole-brain connectivity matrix and the vectorised tract matrix. The columns of this matrix give the cortical termination patterns of each tract, whereas the rows provide the connectivity pattern of each of the cortical locations, as illustrated in Fig. 9. Using this approach, we constructed connectivity blueprints for the neonatal, adult, and macaque brain using WGB surfaces.

#### Surface extraction

Neonatal cortical surfaces were reconstructed from T2-weighted images, using a pipeline specifically adapted for neonatal structural MRI data (*79*). These surfaces were registered to a representative template space before performing tractography, to ensure alignment between subjects. Subjects’ WGB surfaces were first aligned to the dHCP’s 40-week PMA surface template (*80*), using a specialized surface registration pipeline (https://github.com/ecr05/dHCP_template_alignment), based on multimodal surface matching (MSM) (*81*). This aligned vertices on the WGB to ensure consistent seed points for tractography across subjects. A previously computed nonlinear volumetric registration (*82*) was then applied to all MSM-derived surfaces to register them to 40-week PMA volumetric template space (*68*). This step was necessary to ensure that the tractography seeds were aligned to the target space.

The adult surfaces were derived using the HCP pipelines (*83*). For the macaque surface data, we follow the approach of (*8*). Briefly, a single set of macaque surfaces were derived using a set of high-quality structural data from one of the macaque subjects. The remaining macaque data were then nonlinearly transformed to this space, and the surfaces were nonlinearly transformed to the F99 standard space (*75*) to allow group-level tractography.

Before tractography, the surfaces were downsampled to approximately 10,000 vertices per hemisphere. We then carried out probabilistic tractography, seeding 1000 streamlines from each vertex on the WGB, and recording visitation counts between each seed point and each voxel in a whole-brain mask with the ventricles removed, down-sampled to 2 mm^3^ for the neonatal and macaque brain and 3 mm^3^ for adult brain.

#### Group-averaged blueprints

Following subject-wise construction of connectivity blueprints, we derived group-averaged blueprints for each dataset. Average connectivity blueprints were generated using 33 full-term neonates born and scanned just after birth (at 40 weeks PMA) from the dHCP cohort, 50 adult subjects from the HCP cohort and 6 macaques. Further group-averaged connectivity blueprints were derived for other subgroups of the neonatal dHCP cohort: a group of 25 very premature infants (<32 weeks of gestational age at birth) who were scanned at full-term equivalence (37 to 45 weeks), a sex- and age (at scan)–matched group of 25 neonates born full-term and also generated for three groups of 73 full-term neonates scanned at three age ranges (36 to 40, 40 to 42, and 42 to 45 weeks). For full subgroup details, see fig. S9.

### Connectivity embedding for comparing connections with adult humans and macaques

Connectivity patterns, as captured by rows of the connectivity blueprints, were compared across age groups and species, using KL divergence (Eq. 1) (*19*). Let *N* be a neonatal connectivity blueprint matrix and *N_ik_* represent the likelihood of a connection from vertex *i* on the neonatal cortex to tract *k*. Let matrix *A* be the equivalent matrix for the adult brain, with the same number of tracts *T*. Vertices *i* and *j* in the neonatal and adult brains can then be compared in terms of their connectivity patterns {*N_ik_*, *A_jk_*, *k* = 1:*T*} using the symmetric KL divergence *D_ij_* as a dissimilarity measure

$$D_{ij}=\sum_{k}N_{ik}{\text{log}}_{2}\frac{N_{ik}}{A_{jk}}+\sum_{k}A_{jk}{\text{log}}_{2}\frac{A_{jk}}{N_{ik}}$$ (1)

The same process can be used to compare any two brains *N* and *A* (e.g., human with that of the macaque). This provides a matrix describing the (dis-)similarity between each of the cortical locations across the compared brains. The closest matching cortical locations across brains may be revealed by minimizing the KL divergence, i.e., arg min(**d**), where **d** is the *i*th row vector of *D_ij_*.

#### Parcellated divergence

When generating parcellated KL divergence matrices, first the KL divergence was calculated between the two dense (i.e., vertex-wise) connectivity blueprints, and the KL divergence matrix was subsequently parcellated (parcel-wise median) along columns and rows using the relevant cortical parcellation scheme. The neonatal KL divergence data were parcellated using the Melbourne Children’s Regional Infant Brain (MCRIB-S) neonatal parcellation, which is compatible with the DK parcellation. For the adult human, we used the DK cortical atlas, as well as the standard Brodmann cortical atlas. We used the Brodmann vervet monkey atlas for the macaque data. In all cases, we excluded the insula (Brodmann areas 13, 14, and 16 and insula in DK), as it was poorly represented by the set of tracts reconstructed, and we also excluded Brodmann area 26 due to its very small size in the human brain.

As before, once parcellated, the minimum KL divergence between parcels may be found. Alternatively, as used in the divergence against neonatal age and preterm/full-term analyses, where both the neonate and adult brains were parcellated using the DK cortical atlas, the KL divergence between corresponding parcels may be obtained by taking the diagonal of the parcellated KL divergence matrix diag(*D_ij_*).

#### Bootstrapping

To explore the stability of the ontogeny-phylogeny KL divergence maps, we performed a bootstrapped analysis, repeatedly calculating the KL divergence between (i) the macaque and neonate, (ii) the neonate and adult, and (iii) the macaque and adult. We randomly subsampled (with replacement) each of the three datasets over 100 iterations, using 20, 20, and 4 subjects from the neonate (from the pool of 33 neonates in the 40-week group), adult, and macaque datasets, respectively. In each iteration, we averaged the connectivity blueprints for each subsample and calculated the KL divergence maps to report the mean and variance across iterations.

#### Connectivity embedding

Using connectivity blueprints as a common connectivity space, we may translate and compare cortical atlases across diverse brains. Following the approach introduced in (*8*), we projected parcellated KL divergence matrices to a low-dimensional space using spectral embedding (*31*). Spectral embedding groups parcels with similar connectivity profiles together in the projected space. Through this, we compared connectivity within and across cortical atlases between the neonatal and adult brain and the macaque and adult brain.

## Supplementary Materials

### This PDF file includes:

Supplementary Text Figs. S1 to S9 Tables S1 and S2 References

## Notes

View/request a protocol for this paper from *Bio-protocol*.
