# Supplementary material for: Concurrent mapping of brain ontogeny and phylogeny within a common space: Standardized tractography and applications
Source: Sci Adv. 2022 Oct 19;8(42):eabq2022. doi: 10.1126/sciadv.abq2022 (PMC9581484; doi:10.1126/sciadv.abq2022)
Supplement: Supplementary file 1 — Supplementary Text Figs. S1 to S9 Tables S1 and S2 References [file sciadv.abq2022_sm.pdf]

Science Advances
AAAS

Supplementary Materials for

Concurrent mapping of brain ontogeny and phylogeny within a common space: Standardized tractography and applications

Shaun Warrington *et al.*

Corresponding author: Shaun Warrington, shaun.warrington1@nottingham.ac.uk;
Stamatios N. Sotiropoulos, stamatios.sotiropoulos@nottingham.ac.uk

Sci. Adv. **8**, eabq2022 (2022)
DOI: 10.1126/sciadv.abq2022

This PDF file includes:

Supplementary Text
Figs. S1 to S9
Tables S1 and S2
References

Supplementary Text

Tractography (baby-XTRACT) Protocol Definitions

The following protocols were previously described in (*12*). For completeness, we reproduce them here and highlight any changes compared to the adult human XTRACT protocols.

Association Fibres

Arcuate Fasciculus (AF)

The arcuate fasciculus is associated with language functioning (*20*). Compared to the original XTRACT protocols, the ordering of the seed and target masks were exchanged, as this was found to promote more frontal projections of the tract. A seed mask was placed at the level of the ventral premotor cortex, posterior to the inferior frontal gyrus (IFG) and anterior to the precentral sulcus. Two target masks were used, first in the supramarginal gyrus (SMG) and a second temporal target mask was in the WM encompassing the superior temporal gyrus (STG) and middle temporal gyrus (MTG). Streamlines were constrained to pass through the targets in this order. An axial exclusion mask was placed at the level of the cingulate sulcus, and another axial exclusion was placed in the subcortical grey matter to prevent leakage into the internal capsule. A final exclusion mask was added to the lateral sulcus to prevent erroneous streamlines crossing between the gyri.

Frontal Aslant Tract (FA)

The frontal aslant tract is a short bundle running in the frontal lobe between the posterior part of the inferior and superior frontal gyri (*84*). The seed was placed sagittally in the white matter of the IFG, and the target was placed axially in the white matter of the SFG. A posterior exclusion mask prevented leakage into longitudinal fibres.

Inferior Fronto-Occipital Fasciculus (IFO)

The inferior fronto-occipital fasciculus courses along the temporal lobe, passing through the extreme capsule before reaching the frontal cortex. As in the adult protocols, the seed was a coronal plane through the anterior part of the occipital cortex, the target a coronal plane through the frontal cortex, anterior to the genu of the corpus callosum. An exclusion mask just behind the anterior commissure excluded all fibres except those running through the extreme capsule.

Inferior/Middle Longitudinal Fasciculus (ILF, MdLF)

The inferior and middle longitudinal fasciculi are tracts within the lateral posterior cortex of the temporal lobe. The MdLF was seeded in the anterior part of the superior frontal gyrus (SFG) (*85*), and the ILF in the middle and inferior temporal gyri. For the MdLF, large axial and coronal planes covering the white matter in the temporo-parietal-occipital junction, were used as targets. For the ILF, a coronal plane in the middle and inferior temporal gyrus is used as a target. For both protocols, exclusion masks were placed axially through the brainstem, coronally through the fornix, axially through the cingulum bundle posterior to the corpus callosum and through the entire frontal cortex. In addition, the seed mask of the MdLF served as an exclusion mask for the ILF and vice versa, and the ILF target mask was used as an exclusion mask for the MdLF. Additionally, for the ILF, a coronal exclusion mask was placed in the centrum semiovale and an axial exclusion mask covering the white matter of the SMG was used.

Superior Longitudinal Fasciculus (SLF) 1/2/3

The superior longitudinal fasciculus is a longitudinal parieto-frontal tract associated with visuospatial attention (*56*). It can be separated into three bundles: a dorsal superior longitudinal fasciculus SLF1, middle SLF2 and ventral SLF3. For each bundle, a coronal plane in the region of the central sulcus within the frontoparietal cortex is used as a seed, along with two target masks. The frontal target masks for the first, second and third branches of the SLF were coronal sections through the superior, middle and inferior frontal gyri, respectively, placed at the level of the posterior end of the corpus callosum. Posteriorly, a large coronal target mask in the superior parietal lobule, immediately posterior to the margin of the cingulate gyrus is used for SLF1. For SLF2 and SLF3, the second target masks are placed in the angular gyrus and supramarginal gyrus respectively. In each case, seed placement reflects the placement of the second target whilst being moved anteriorly into the region of the central sulcus. For each protocol, an axial exclusion mask was placed underneath the parietal cortex, and one blocking subcortical areas prevented leakage into ventrally oriented fibres. A coronal exclusion mask through subcortical areas posterior to the caudal end of the genu of the corpus callosum prevented streamlines leaking into ventral longitudinal tracts. The SLFs are still maturing during the neonatal period, which makes them challenging to delineate in this age-group (*86*). Therefore, extra exclusion masks were added compared to the adult protocols, to help constrain the tractography. For each branch, the target masks for each of the other SLF branches were included as exclusion masks. An additional exclusion mask was also placed in the cingulate gyrus for the SLF1, to prevent leakage into this region. The seed and target masks were also increased in size relative to the anatomy, compared to the adult masks, which made the results more robust.

Uncinate Fasciculus (UF)

The uncinate fasciculus is a hook-shaped bundle that connects the frontal lobe with the anterior temporal lobe. The tract was reconstructed using a seed in the STG at the first location where the temporal and frontal cortex are separated, and a target through the ventral part of the extreme capsule. An exclusion mask layer was added between the seed and the target to force the streamlines to curve between them. A coronal exclusion mask prevented leakage into the fibres running longitudinally through the temporal lobe. For the neonatal protocols, an additional axial exclusion mask was added to prevent fibres leaking into the external capsule.

Vertical Occipital Fasciculus (VOF)

The vertical occipital fasciculus runs in a predominantly dorsal-ventral orientation in the occipital lobe. The original protocol was adapted from ref. (*87*). An axial seed mask was placed in the lateral part of the ventral occipital white matter posterior to the anterior occipital sulcus. A larger anterior target mask was placed dorsally at the level of the lateral occipital sulcus. A coronal plane just posterior to the corpus callosum served as an exclusion mask, to prevent leakage into anterior-posterior tracts.

Commissural Fibres

Anterior Commissure (AC)

The anterior commissure connects the temporal lobes of the two hemispheres across the midline. It was seeded in the left-right oriented fibres on the midline, with a target mask covering the white matter lateral to the globus pallidae. Stop masks were placed directly underneath and lateral to the two amygdalae. A large axial exclusion mask was placed dorsal to the seed through the entire

subcortex. For the neonatal protocols, an axial exclusion mask was added covering the optic chiasm to prevent leakage into the optic nerve. A further coronal axial exclusion mask was added in the anterior limb of the internal capsule, to prevent erroneous frontal projections from the seed.

Corpus Callosum, Splenium (FMA) and Genu (FMI)
Callosal connections to the occipital lobe were constructed via the splenium of the corpus callosum (forceps major, FMA) and to the frontal lobe via the genu of the corpus callosum (forceps minor, FMI), using recipes based on those defined by ref. (*88*). Seed and target masks, and their inverse, for the FMA were defined as coronal sections through the occipital lobe at the posterior end of the parietal occipital sulcus. The sagittal exclusion mask was confined to the occipital cortex and the subcortex. Additional exclusion masks through the inferior fronto-occipital white matter and a coronal plane through the pons prevented leakage into the longitudinal fibres. Seed and target masks (and their inverse) for the FMI were defined as coronal sections through the frontal lobe at the anterior end of the pregenual cingulate sulcus. The midsagittal exclusion mask was interrupted at the level of the anterior third of the corpus callosum, and an additional coronal exclusion mask at the same level prevents posterior projections.

Middle Cerebella Peduncle (MCP)
The middle cerebellar peduncle connects the cerebellum to the pons. This tract was seeded in the cerebellar white matter with a target in the opposite hemisphere (and their inverses). Exclusion masks were placed sagittally along the cerebellar midline and axially through the thalamus.

Limbic Fibres

Cingulum Subsections (CBT, CBP, CBD)
The cingulum facilitates communication between different parts of the limbic system. It projects from the cingulate gyrus to the entorhinal cortex (*89*). Protocols were defined for three distinct sections of the cingulum, based on a recent segmentation by ref. (*88*). The temporal part (CBT) was seeded in the posterior part of the temporal lobe at a section where the fibres of the cingulum are mostly oriented in the anterior-posterior direction. The target was placed posteriorly to the amygdala. Stop masks were placed posteriorly and anteriorly to the seed and target masks, respectively. An exclusion mask prevented leakage into the fornix. The dorsal segment (CBD) was seeded just above the posterior part of the corpus callosum and had a target at the start of the genu of the corpus callosum. A sagittal exclusion mask in the anterior limb of the internal capsule prevented leakage into the temporal lobe. Finally, the peri-genual part of the cingulum bundle (CBP) was seeded anteriorly above the corpus callosum and a target placed below the sub-genual callosum with a stop mask placed inferior and anterior to the target. A callosal plane at the level of the rostral end of the Sylvian fissure prevented leakage into the CBD.

Fornix (FX)
The fornix connects the hippocampus with the mammillary bodies, the anterior thalamic nuclei, and the hypothalamus (*91*). The tract was reconstructed using a seed in the body of the fornix at the level of the middle of the corpus callosum and a target in the hippocampus. A callosal plane at the anterior end of the occipital cortex prevented leakage into posterior tracts. To prevent leakage into the cingulum, an axial exclusion mask posterior to the splenium of the corpus callosum, and a small axial exclusion covering the parahippocampal gyrus region of the cingulum are also used. The adult protocol contains bilateral sagittal planes around the midline, at the level of the anterior

tip of the thalamus, to prevent lateral propagation to the anterior limb of the internal capsule. These exclusion masks were reduced in size for the neonatal protocol, relative to the adult protocol, as they were found to hinder fibre tracking in the neonates.

Projection Fibres

Acoustic Radiation (AR)

The acoustic radiation connects the medial geniculate nucleus (MGN) of the thalamus to the auditory cortex. It was seeded from the transverse temporal gyrus with a target covering the MGN of the thalamus. The exclusion mask consists of two coronal planes, anterior and posterior to the thalamus, as well as a mask covering the brainstem and a horizontal region covering the optic tract. The adult protocol contains an axial plane superior to the thalamus as an exclusion mask. This mask was moved inferiorly for the neonatal protocols, to stop leakage into the internal capsule.

Anterior Thalamic Radiation (ATR)

The anterior thalamic radiation connects the thalamus to the frontal lobe. It is seeded using a coronal mask through the anterior part of the thalamus (*88*), with a coronal target mask at the anterior thalamic peduncle. In addition, the exclusion mask contains an axial plane covering the base of the midbrain, a coronal plane preventing leakage via the posterior thalamic peduncle and a coronal plane preventing leakage via the cingulum. A coronal stop mask covers the posterior part of the thalamus, extending from the base of the midbrain to the callosal sulcus. An additional axial exclusion mask was added compared to the adult protocol at the level of the cingulate gyrus, to prevent contamination from fibres in the superior thalamic radiation.

Corticospinal tract (CST)

The corticospinal, or pyramidal, tract extends from the spinal cord through the midbrain and distributes to motor cortex, premotor cortex and somatosensory cortex. The tracts is seeded from the pons with a large target covering the motor, premotor and somatosensory cortices. An axial exclusion mask us used to restrict tracking to the cerebral peduncle of the midbrain. In addition, the exclusion mask includes two coronal planes, anterior and posterior to the target, to exclude tracking to the prefrontal cortex and occipital cortex respectively, and a plane preventing leakage into the cerebellar peduncles. To prevent contamination from the spino-thalamic tract, an additional exclusion mask was added in the neonatal protocol, in the posterior part of the brainstem.

Optic Radiation (OR)

The optic radiation consists of fibres connecting the lateral geniculate nucleus (LGN) of the thalamus to the primary visual cortex. It was seeded in the LGN, with a target mask through the anterior part of the calcarine fissure. Exclusion masks consisted of an axial block in the brainstem, a coronal plane directly posterior to the LGN to select fibres that curl around dorsally, and a coronal plane anterior to the seed to prevent leakage into longitudinal fibres. In addition, a further axial exclusion mask was added in the neonatal protocols, anterior to the target mask, to restrict fibres travelling to the parietal lobe.

Superior Thalamic Radiation (STR)

The superior thalamic radiation connects the thalamus to the pre-/post-central gyrus. It was seeded using a mask covering the whole thalamus and an axial target plane covering the superior thalamic

peduncle. An axial plane is used as a stop mask ventrally to the thalamus. The exclusion mask includes two coronal planes anterior and posterior to the target, to exclude tracking to the prefrontal cortex and occipital cortex, respectively.

Supplementary Figures

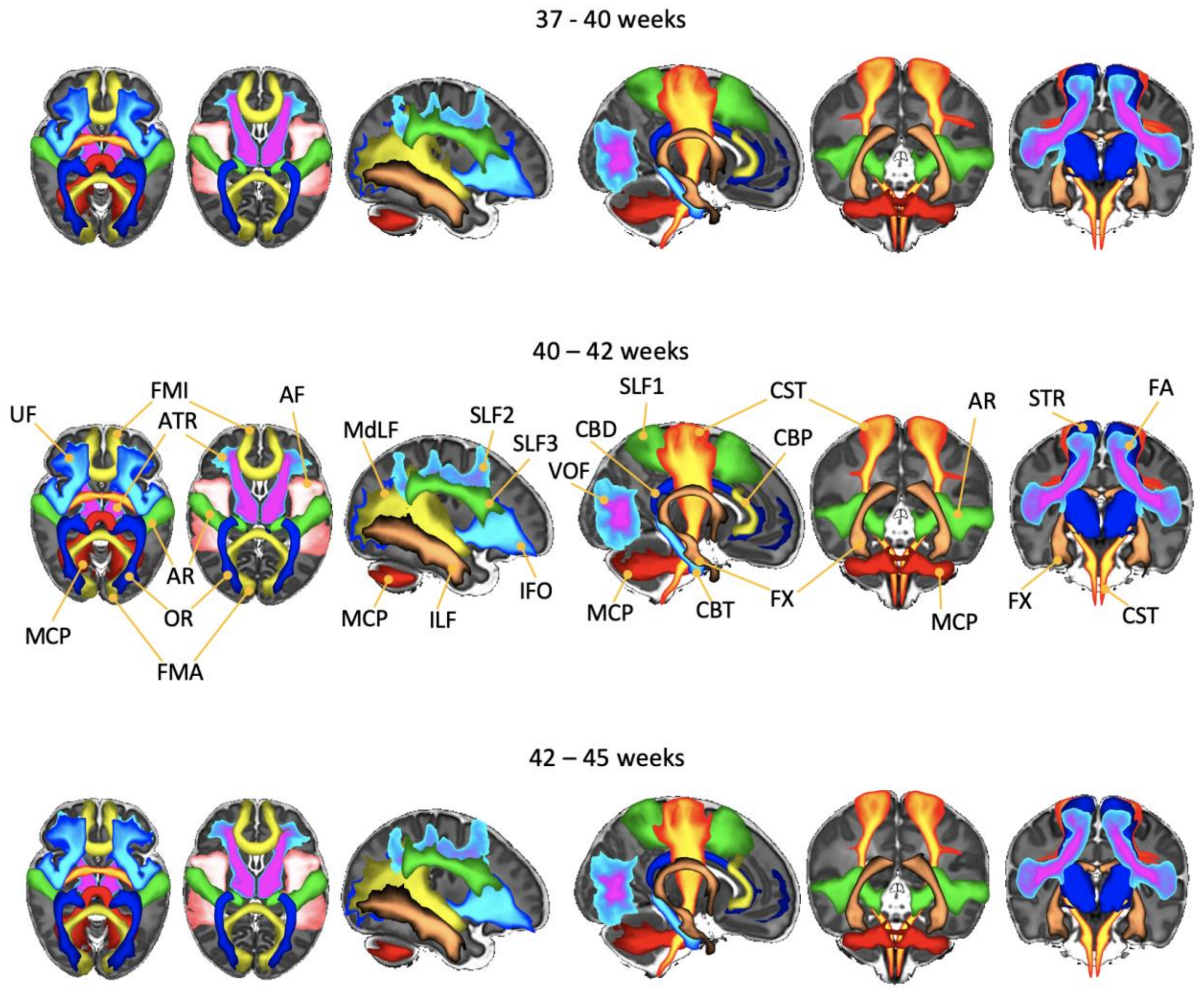

Fig. S1. Neonatal tract atlases show that tractography protocols are robust when applied to neonates at different stages of development. *Neonatal tract atlases (population percentage) for different age groups within the dHCP cohort, with 73 neonates in each sub-group. Top: 37-40 weeks PMA. Middle: 40-42 weeks PMA. Bottom: 42-45 weeks PMA. For display purposes, tracts are displayed as maximum intensity projections, with 30 – 100% population coverage.*

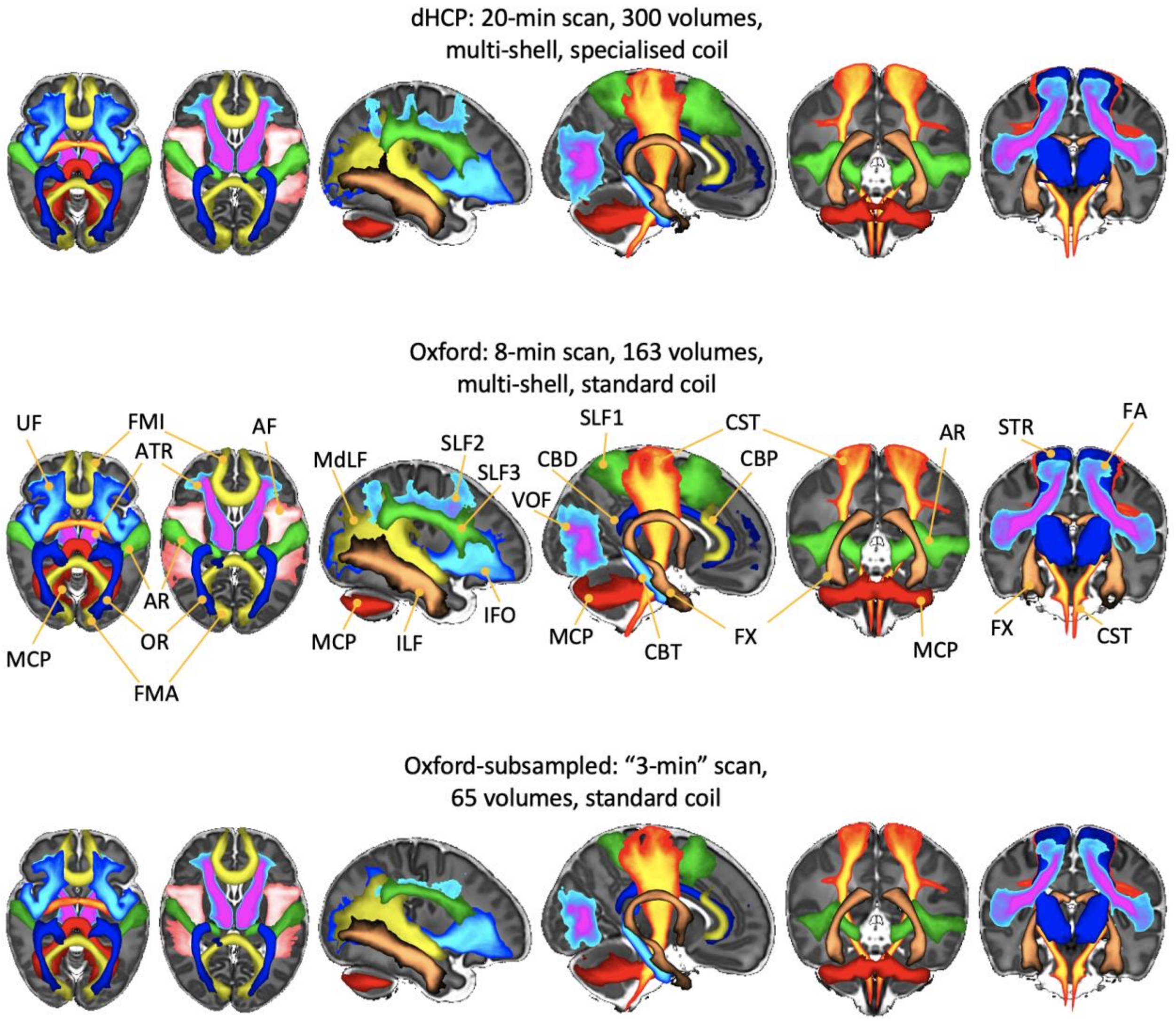

Fig. S2. Neonatal tract atlases show that tractography protocols are robust when applied to data of varying quality. *Top: atlases (population percentage) derived from a group-matched subset of 22 dHCP neonates. Middle: atlases derived from the Oxford dataset. Bottom: atlases derived from the Oxford-subsampled dataset. For display purposes, tracts are displayed as maximum intensity projections, with 30 – 100% population coverage.*

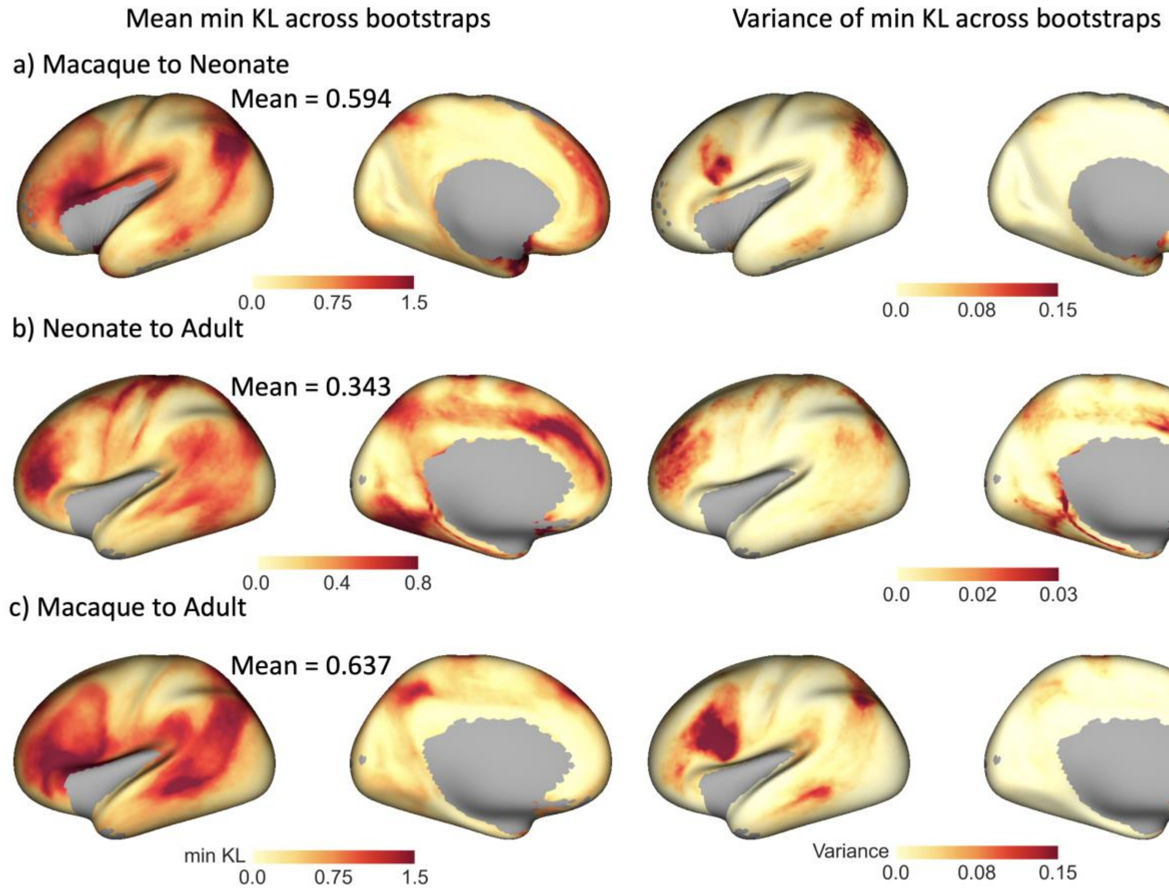

Fig. S3. Bootstrap analysis of ontogeny/phylogeny divergence maps. *The minimum KL divergence maps were repeatedly calculated between a) the macaque and neonate, b) the neonate and adult, and c) the macaque and adult, as in the main text (Fig. 5) using 100 random subsets of the data. Bootstrap samples were constructed by randomly selecting with replacement 20 neonates from the pool of 33 neonates in the 40w age group, 20 random HCP subjects from the pool of 50 subjects and 4 random macaques from the pool of 6. Left: the mean minimum KL divergence maps across 100 bootstrap iterations. Right: the variance in the minimum KL divergence maps across the iterations.*

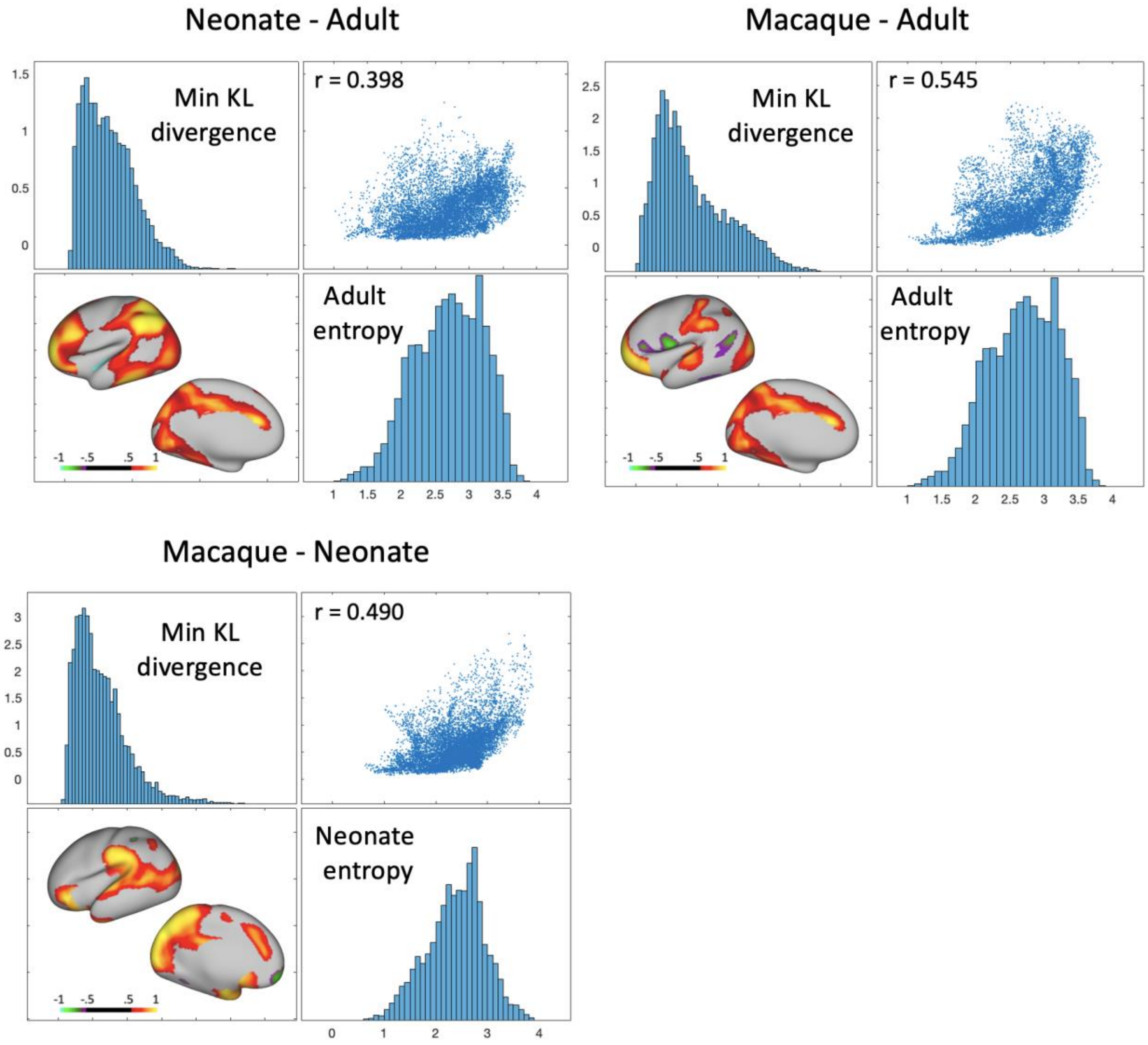

Fig. S4. Association between phylogeny/ontogeny divergence and connectivity profile entropy. *Connectivity profile complexity was calculated at each cortical vertex* $i = 1{:}N$, *as the entropy of the profile (*$H_i = -\sum_{t\in T} X_{it} \log X_{it}$, X *is the connectivity blueprint and* T *the set of tracts). The minimum KL divergence maps presented in Fig. 5(a-c) were compared to these entropy maps through brain-wide and local (i.e. spatially-moving windowed) correlation. The top left and bottom right plots show the distribution of values for the divergence and entropy (of the target brain). The upper right scatter plots show the relationship between all vertices of the divergence and entropy maps and bottom left surface maps show the local correlations. Top left: the neonate-adult divergence map compared to the adult entropy map. Top right: the macaque-adult divergence map compared to the adult entropy map. Bottom left: the macaque-neonate divergence map compared to the neonate entropy map.*

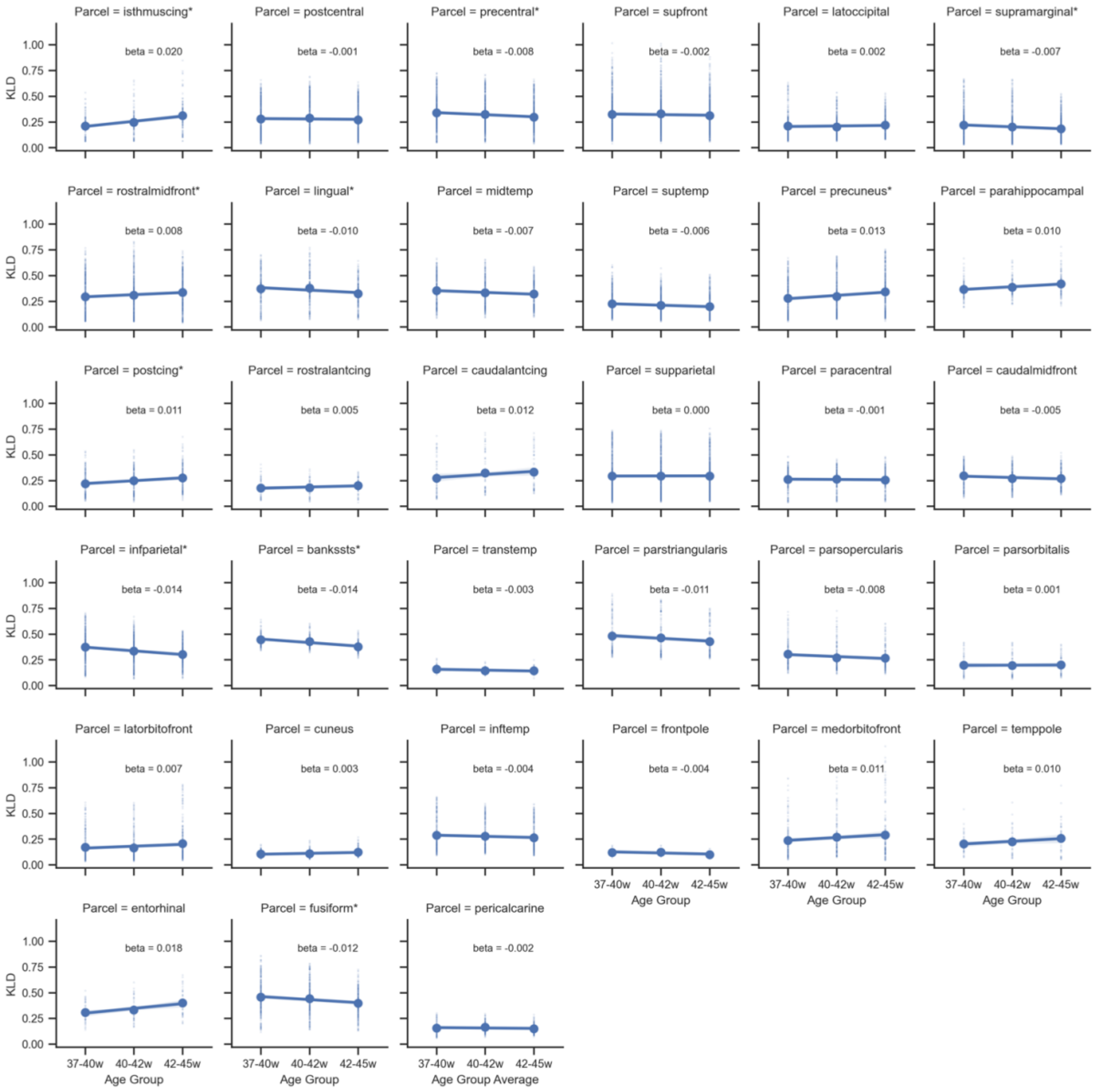

Fig. S5. Parcel-wise changes in dissimilarity (KL divergence) across neonatal age with respect to the adult brain for each Desikan-Killiany parcel. *73 neonates were used to construct averaged connectivity blueprints at each age range which are then compared via KL divergence to the adult connectivity blueprint (averaged over 50 random HCP subjects). The KL divergence between corresponding parcels was calculated and plotted against age. Beta values correspond to the rate of change in KL divergence with neonate age derived via linear regression and the parcel-wise mean is indicated by the large dot. * indicates significant trends following correction for multiple comparisons (p<0.001/32=3.125e-05).*

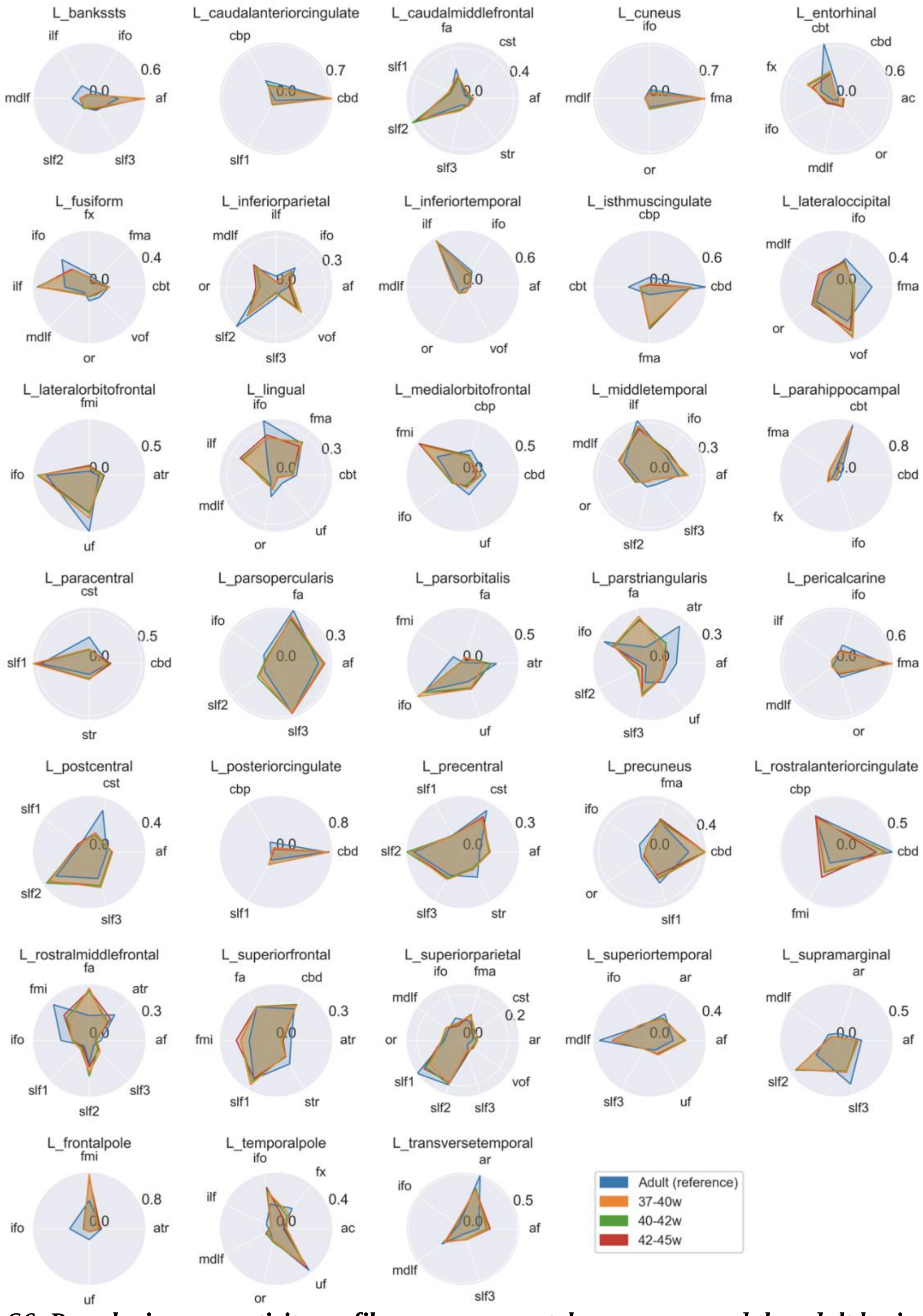

Fig. S6. Parcel-wise connectivity profiles across neonatal age groups and the adult brain. *For visualisation, only the most highly contributing tracts are displayed (tract contribution > 0.05 to any group) and each plot area has been sum-normalised.*

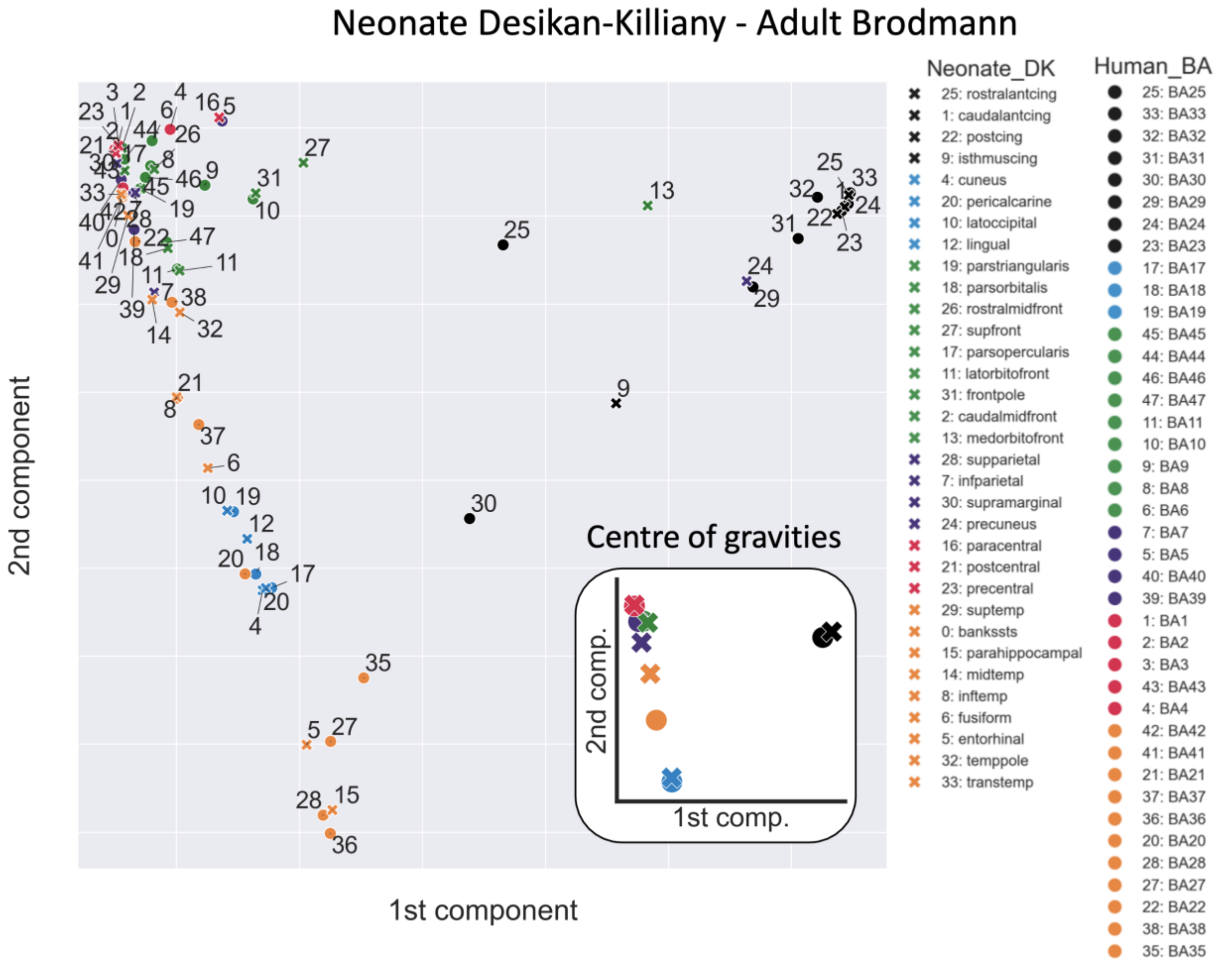

Fig. S7. Larger version of the Neonate-Adult embedding plot (Fig. 8b left). *KL divergence matrices were calculated between group (neonate and adult) connectivity blueprints. Divergence matrices were then parcellated using the Brodmann cortical atlas for the adult brain and the Desikan-Killany cortical atlas for the neonatal brain, taking the median value for each region. We further define a set of anatomo-functional cortical systems (see Fig. 8). The inverse of the KL divergence matrices (i.e. the similarity) was then used as a feature set in spectral embedding and the first two components projected into a 2-dimensional space, colour-coded by major brain regions. Circles represent the Brodmann parcels for the adult brain and crosses represent the Desikan-Killany parcels for the neonate brain. Figure insets show the centre of gravity (median of parcel coordinates) for each anatomo-functionally defined cortical system for each brain. Legend key: "cing" = cingulate; "front" = frontal; "temp" = temporal; "trans" = transverse; "med" = medial; "mid" = "middle"; "inf" = inferior; "lat" = lateral; "ant" = anterior; "post" = posterior.*

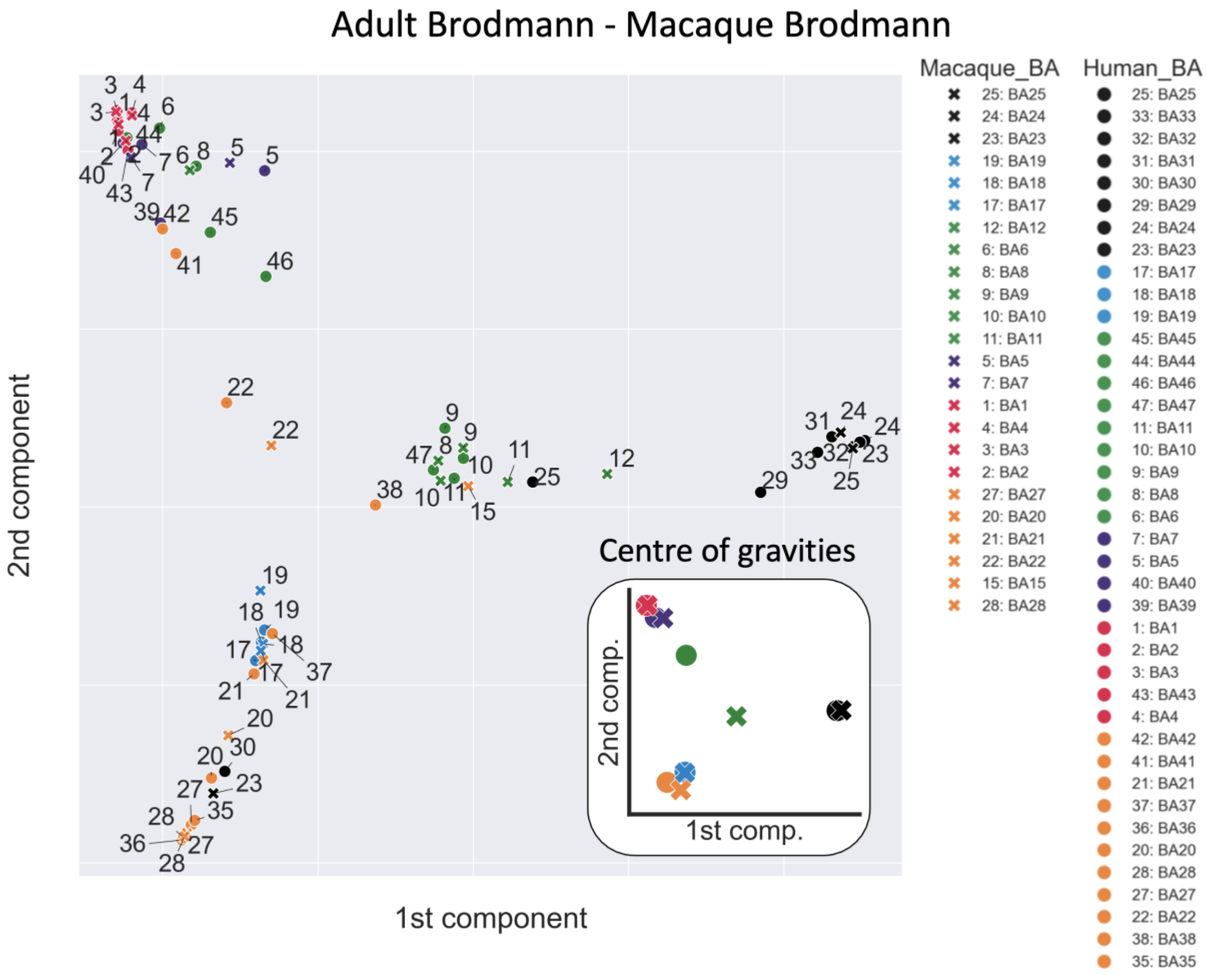

Fig. S8. Larger version of the Macaque-Adult embedding plot (Fig. 8b right). *As above but now between the adult human and macaque brain, parcellated using the Brodmann cortical atlas. Circles represent the Brodmann parcels for the adult brain and crosses represent the Brodmann parcels for the macaque brain.*

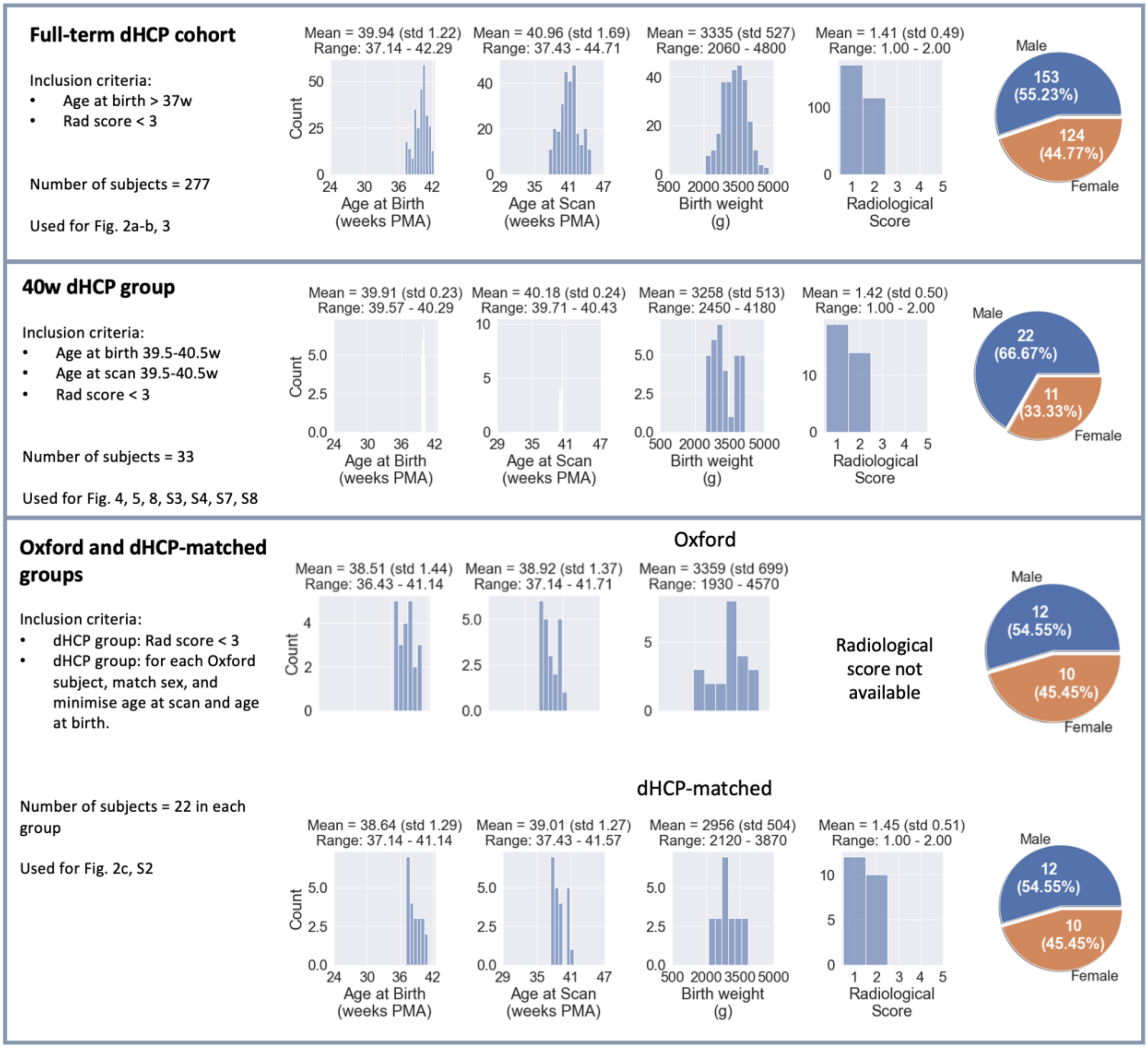

Fig. S9a. Demographics for the dHCP cohort subgroups, and the Oxford cohort. *Top row: the full-term, normally-appearing cohort used for the tract maturation analysis and in the generation of high-quality tract atlases. Middle row: a group of neonates born and scanned at approximately 40w PMA used in ontogeny-phylogeny explorations. Bottom row: The Oxford and dHCP subgroups used to assess protocol robustness against data quality. For each Oxford subject, an age (scan and birth) and sex matched subject is selected from the full dHCP cohort.*

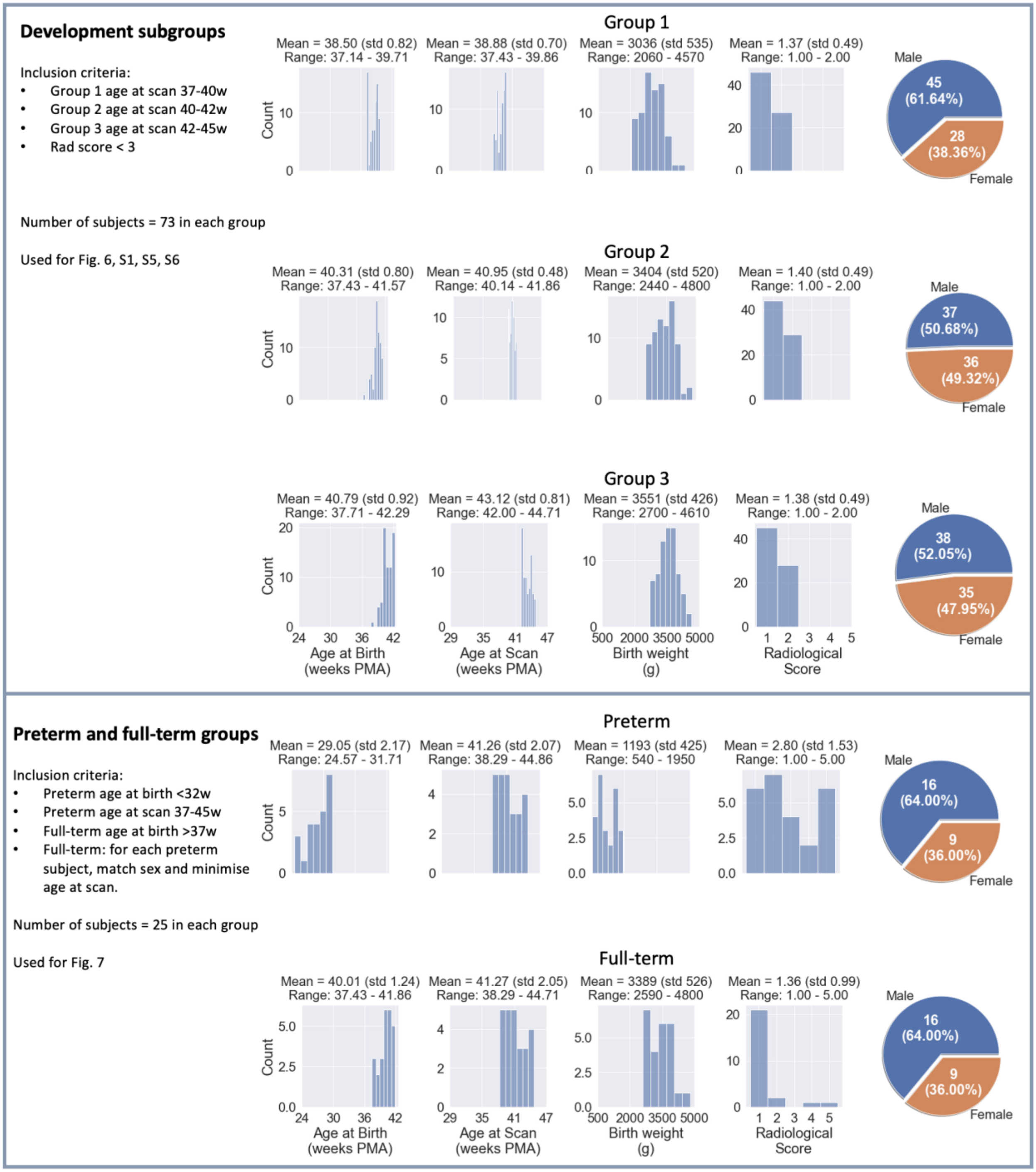

Fig. S9b. Continued demographics for the dHCP cohort subgroups. *Top row: the three developmental age-groups (37-40w, 40-42w, and 42-45w postmenstrual age (PMA)) used to assess protocol robustness across neonate age groups and to explore how divergence, with respect to the adult brain, changes with neonatal age - the number of subjects in each group is restricted to the minimum in any group. Bottom row: groups of premature and full-term neonates used to explore the effects of prematurity – the premature group is selected based on age at birth (<37w) and age at scan (37-45w) and full-term neonates are sub-selected from the full dHCP cohort to match for age at scan and sex.*

		dHCP	Oxford	Oxford-subsampled
Hardware	Scanner	3T Philips Achieva	3T Siemens Prisma	3T Siemens Prisma
	Head coil	32-channel neonatal	32-channel adult	32-channel adult
Acquisition protocol	TE (ms)	90	73	73
	TR (ms)	3800	2900	2900
	Multiband factor	4	3	3
	Spatial resolution (isotropic) (mm)	1.5	1.75	1.75
	# volumes	300	163	65
	b-values (s/mm^2)	0, 400, 1000, 2600	0, 500 ,1000, 2000	0, 500, 1000
	# volumes per b	20, 64, 88, 128	20, 23, 50, 70	5, 10, 50
	Acquisition time (approx. minutes)	20	8	3
Image quality and modelling	Effective SNR/CNR for each b-value	17.12, 1.02, 1.96, 1.97	16.97, 0.75, 1.83, 1.63	8.43, 0.49, 1.83
	% of WM voxels with 2, 3 fibre orientations	59%, 6%	60%, 10%	53%, 3%

Table S1. Description of the three neonatal datasets used in this study. *The Oxford dataset is from ref. (78). This dataset was further subsampled to generate a third dataset (the "Oxford-subsampled" dataset) without the b=2000 s/mm^2 shell, which could be acquired in more clinically feasible scan times. Effective SNR (for b-value = 0) and CNR allows for fair comparison of SNR/CNR across datasets and is calculated as the SNR/CNR (output from eddy QC) divided by voxel volume and multiplied by the square root of the number of volumes collected for a given b-value (71, 92). The percent of WM voxels with 2/3 fibre orientations is calculated by counting the number of voxels (within a WM mask) where the data supports 2/3 fibre orientations, and converting this value to a percentage (notice that the Oxford data have slightly higher number of crossings, but has 60% larger voxels than dHCP). SNR/CNR values and the percent of WM voxels with 2/3 fibre orientations are the average across all 22 subjects.*

		HCP (adult human)	Macaque
**Hardware**	**Scanner**	3T Siemens Connectom Skyra	7T Agilent Technologies DirectDrive
	**Head coil**	32-channel adult	72mm ID quadrature birdcage
**Acquisition protocol**	**Sequence type**	Stejskal-Tanner SE EPI	DW-SEMS single-line readout
	**TE (ms)**	89.5	25
	**TR (ms)**	5520	10000
	**Multiband factor**	3	-
	**Spatial resolution (isotropic) (mm)**	1.25	0.6
	**# volumes**	276	144
	**b-values (s/mm^2)**	0, 1000, 2000, 3000	0, 4000
	**# volumes per b**	6, 90, 90, 90	16, 128
	**Acquisition time (approx.)**	60 minutes	53 hours

Table S2. Acquisition details for the adult human and macaque data. Full acquisition details are available from the Human Connectome Project (HCP) (humanconnectome.org/hcp-protocols-ya-3t-imaging) and PRIME-DE database (fcon_1000.projects.nitrc.org/indi/PRIME/oxford2.html) for the human and macaque data respectively.

REFERENCES AND NOTES

1. D. C. Van Essen, C. J. Donahue, M. F. Glasser, Development and evolution of cerebral and cerebellar cortex. *Brain Behav. Evol.* **91**, 158–169 (2018).

2. J. Hill, T. Inder, J. Neil, D. Dierker, J. Harwell, D. Van Essen, Similar patterns of cortical expansion during human development and evolution*Proc. Natl. Acad. Sci. U.S.A.* **107**, 13135–13140 2010.

3. R. E. Passingham, K. E. Stephan, R. Kötter, The anatomical basis of functional localization in the cortex. *Nat. Rev. Neurosci.* **3**, 606–616 (2002).

4. K. Heuer, O. F. Gulban, P.-L. Bazin, A. Osoianu, R. Valabregue, M. Santin, M. Herbin, R. Toro, Evolution of neocortical folding: A phylogenetic comparative analysis of MRI from 34 primate species. *Cortex* **118**, 275–291 (2019).

5. M. F. Glasser, T. S. Coalson, E. C. Robinson, C. D. Hacker, J. Harwell, E. Yacoub, K. Ugurbil, J. Andersson, C. F. Beckmann, M. Jenkinson, S. M. Smith, D. C. Van Essen, A multi-modal parcellation of human cerebral cortex. *Nature* **536**, 171–178 (2016).

6. D. C. Van Essen, Cartography and connectomes. *Neuron* **80**, 775–790 (2013).

7. T. Xu, K.-H. Nenning, E. Schwartz, S.-J. Hong, J. T. Vogelstein, A. Goulas, D. A. Fair, C. E. Schroeder, D. S. Margulies, J. Smallwood, M. P. Milham, G. Langs, Cross-species functional alignment reveals evolutionary hierarchy within the connectome. *NeuroImage* **223**, 117346 (2020).

8. R. B. Mars, S. N. Sotiropoulos, R. E. Passingham, J. Sallet, L. Verhagen, A. A. Krapitchev, N. Sibson, J. Saad, Whole brain comparative anatomy using connectivity blueprints. *Elife* **7**, e35237 (2018).

9. N. Eichert, E. C. Robinson, K. L. Bryant, S. Jbabdi, M. Jenkinson, L. Li, K. Krug, K. E. Watkins, R. B. Mars, Cross-species cortical alignment identifies different types of anatomical reorganization in the primate temporal lobe. *Elife* **9**, e53232 (2020).

10. R. B. Mars, R. E. Passingham, S. Jbabdi, Connectivity fingerprints: From areal descriptions to abstract spaces. *Trends Cogn. Sci.* **22**, 1026–1037 (2018).

11. E. Thompson, A. R. Mohammadi-Nejad, E. C. Robinson, J. L. R. Andersson, S. Jbabdi, M. F. Glasser, M. Bastiani, S. N. Sotiropoulos, Non-negative data-driven mapping of structural connections with application to the neonatal brain. *Neuroimage* **222**, 117273 (2020).

12. S. Warrington, K. L. Bryant, A. A. Khrapitchev, J. Sallet, M. Charquero-Ballester, G. Douaud, S. Jbabdi, R. B. Mars, S. N. Sotiropoulos, XTRACT – Standardised protocols for automated tractography in the human and macaque brain. *Neuroimage* **217**, 116923 (2020).

13. E. J. Hughes, T. Winchman, F. Padormo, R. Teixeira, J. Wurie, M. Sharma, M. Fox, J. Hutter, L. Cordero-Grande, A. N. Price, J. Allsop, J. Bueno-Conde, N. Tusor, T. Arichi, A. D. Edwards, M. A. Rutherford, S. J. Counsell, J. V. Hajnal, A dedicated neonatal brain imaging system. *Magn. Reson. Med.* **78**, 794–804 (2017).

14. J. Dubois, G. Dehaene-Lambertz, M. Perrin, J. F. Mangin, Y. Cointepas, E. Duchesnay, D. Le Bihan, L. Hertz-Pannier, Asynchrony of the early maturation of white matter bundles in healthy infants: Quantitative landmarks revealed noninvasively by diffusion tensor imaging. *Hum. Brain Mapp.* **29**, 14–27 (2008).

15. S. Khan, L. Vasung, B. Marami, C. K. Rollins, O. Afacan, C. M. Ortinau, E. Yang, S. K. Warfield, A. Gholipour, Fetal brain growth portrayed by a spatiotemporal diffusion tensor MRI atlas computed from in utero images. *Neuroimage* **185**, 593–608 (2019).

16. H. Huang, L. Vasung, Gaining insight of fetal brain development with diffusion MRI and histology. *Int. J. Dev. Neurosci.* **32**, 11–22 (2014).

17. J.-D. Tournier, C.-H. Yeh, F. Calamante, K.-H. Cho, A. Connelly, C.-P. Lin, Resolving crossing fibres using constrained spherical deconvolution: Validation using diffusion-weighted imaging phantom data. *Neuroimage* **42**, 617–625 (2008).

18. G. Ball, L. Srinivasan, P. Aljabar, S. J. Counsell, G. Durighel, J. V Hajnal, M. A. Rutherford, A. D. Edwards, Development of cortical microstructure in the preterm human brain. *Proc. Natl. Acad. Sci.* **110**, 9541–9546 (2013).

19. S. Kullback, R. A. Leibler, On information and sufficiency. *Ann. Math. Stat.* **22**, 79–86 (1951).

20. N. Eichert, L. Verhagen, D. Folloni, S. Jbabdi, A. A. Khrapitchev, N. R. Sibson, D. Mantini, J. Sallet, R. B. Mars, What is special about the human arcuate fasciculus? Lateralization, projections, and expansion. *Cortex* **118**, 107–115 (2019).

21. J. K. Rilling, M. F. Glasser, T. M. Preuss, X. Ma, T. Zhao, X. Hu, T. E. J. Behrens, The evolution of the arcuate fasciculus revealed with comparative DTI. *Nat. Neurosci.* **11**, 426–428 (2008).

22. J. Brauer, A. Anwander, D. Perani, A. D. Friederici, Dorsal and ventral pathways in language development. *Brain Lang.* **127**, 289–295 (2013).

23. D. Perani, M. C. Saccuman, P. Scifo, A. Anwander, D. Spada, C. Baldoli, A. Poloniato, G. Lohmann, A. D. Friederici, Neural language networks at birth. *Proc. Natl. Acad. Sci. U.S.A.*, **108**, 16056–16061 (2011).

24. J. Zhao, M. Thiebaut de Schotten, I. Altarelli, J. Dubois, F. Ramus, Altered hemispheric lateralization of white matter pathways in developmental dyslexia: Evidence from spherical deconvolution tractography. *Cortex* **76**, 51–62 (2016).

25. J. F. de Kieviet, L. Zoetebier, R. M. van Elburg, R. J. Vermeulen, J. Oosterlaan, Brain development of very preterm and very low-birthweight children in childhood and adolescence: A meta-analysis. *Dev. Med. Child Neurol.* **54**, 313–323 (2012).

26. S. E. Rose, X. Hatzigeorgiou, M. W. Strudwick, G. Durbridge, P. S. W. Davies, P. B. Colditz, Altered white matter diffusion anisotropy in normal and preterm infants at term-equivalent age. *Magn. Reson. Med.* **60**, 761–767 (2008).

27. M. M. Vandewouw, J. M. Young, M. M. Shroff, M. J. Taylor, J. G. Sled, Altered myelin maturation in four year old children born very preterm. *NeuroImage Clin.* **21**, 101635 (2019).

28. G. Ball, J. P. Boardman, P. Aljabar, A. Pandit, T. Arichi, N. Merchant, D. Rueckert, A. D. Edwards, S. J. Counsell, The influence of preterm birth on the developing thalamocortical connectome. *Cortex* **49**, 1711–1721 (2013).

29. D. C. Van Essen, M. F. Glasser, D. L. Dierker, J. Harwell, Cortical parcellations of the macaque monkey analyzed on surface-based atlases. *Cereb. Cortex* **22**, 2227–2240 (2012).

30. B. Alexander, W. Y. Loh, L. G. Matthews, A. L. Murray, C. Adamson, R. Beare, J. Chen, C. E. Kelly, P. J. Anderson, L. W. Doyle, A. J. Spittle, J. L. Y. Cheong, M. L. Seal, D. K. Thompson, Desikan-Killiany-Tourville atlas compatible version of M-CRIB neonatal parcellated whole brain atlas: The M-CRIB 2.0. *Front. Neurosci.* **13**, 34 (2019).

31. D. J. Higham, G. Kalna, M. Kibble, Spectral clustering and its use in bioinformatics. *J. Comput. Appl. Math.* **204**, 25–37 (2007).

32. S. van Heukelum, R. B. Mars, M. Guthrie, J. K. Buitelaar, C. F. Beckmann, P. H. E. Tiesinga, B. A. Vogt, J. C. Glennon, M. N. Havenith, Where is cingulate cortex? A cross-species view. *Trends Neurosci.* **43**, 285–299 (2020).

33. T. K. Turesky, J. Vanderauwera, N. Gaab, Imaging the rapidly developing brain: Current challenges for MRI studies in the first five years of life. *Dev. Cogn. Neurosci.* **47**, 100893 (2021).

34. M. Laubach, L. M. Amarante, K. Swanson, S. R. White, What, if anything, is rodent prefrontal cortex? *eNeuro* **5**, 10.1523/ENEURO.0315-18.2018 (2018).

35. R. B. Mars, S. Jbabdi, M. F. S. Rushworth, A common space approach to comparative neuroscience. *Annu. Rev. Neurosci.* **44**, 69–86 (2021).

36. K. Akazawa, L. Chang, R. Yamakawa, S. Hayama, S. Buchthal, D. Alicata, T. Andres, D. Castillo, K. Oishi, J. Skranes, T. Ernst, K. Oishi, Probabilistic maps of the white matter tracts

with known associated functions on the neonatal brain atlas: Application to evaluate longitudinal developmental trajectories in term-born and preterm-born infants. *Neuroimage* **128**, 167–179 (2016).

37. D. Anblagan, M. E. Bastin, S. Sparrow, C. Piyasena, R. Pataky, E. J. Moore, A. Serag, A. G. Wilkinson, J. D. Clayden, S. I. Semple, J. P. Boardman, Tract shape modeling detects changes associated with preterm birth and neuroprotective treatment effects. *NeuroImage. Clin.* **8**, 51–58 (2015).

38. L. Zöllei, C. Jaimes, E. Saliba, P. E. Grant, A. Yendiki, Tracts constrained by underlying infant anatomy (TRACULInA): An automated probabilistic tractography tool with anatomical priors for use in the newborn brain. *Neuroimage* **199**, 1–17 (2019).

39. M. Á. García-Cabezas, B. Zikopoulos, Evolution, development, and organization of the cortical connectome. *PLOS Biol.* **17**, e3000259 (2019).

40. K. Teffer, K. Semendeferi, Human prefrontal cortex: Evolution, development, and pathology. *Prog. Brain Res.* **195**, 191–218 (2012).

41. J. Lebenberg, J.-F. Mangin, B. Thirion, C. Poupon, L. Hertz-Pannier, F. Leroy, P. Adibpour, G. Dehaene-Lambertz, J. Dubois, Mapping the asynchrony of cortical maturation in the infant brain: A MRI multi-parametric clustering approach. *Neuroimage* **185**, 641–653 (2019).

42. F.-X. Neubert, R. B. Mars, A. G. Thomas, J. Sallet, M. F. S. Rushworth, Comparison of human ventral frontal cortex areas for cognitive control and language with areas in monkey frontal cortex. *Neuron* **81**, 700–713 (2014).

43. E. Koechlin, Frontal pole function: What is specifically human? *Trends Cogn. Sci.* **15**, 241 (2011).

44. R. B. Mars, S. Jbabdi, J. Sallet, J. X. O'Reilly, P. L. Croxson, E. Olivier, M. P. Noonan, C. Bergmann, A. S. Mitchell, M. G. Baxter, T. E. J. Behrens, H. Johansen-Berg, V. Tomassini, K. L. Miller, M. F. S. Rushworth, Diffusion-weighted imaging tractography-based

parcellation of the human parietal cortex and comparison with human and macaque resting-state functional connectivity. *J. Neurosci.* **31**, 4087–4100 (2011).

45. K. Amunts, K. Zilles, Architecture and organizational principles of Broca's region. *Trends Cogn. Sci.* **16**, 418–426 (2012).

46. S. Heim, S. B. Eickhoff, K. Amunts, Specialisation in Broca's region for semantic, phonological, and syntactic fluency? *Neuroimage* **40**, 1362–1368 (2008).

47. I. Kostović, G. Sedmak, M. Judaš, Neural histology and neurogenesis of the human fetal and infant brain. *Neuroimage* **188**, 743–773 (2019).

48. L. Roumazeilles, Structure and function of the social brain in primates, PhD thesis, University of Oxford (2021).

49. B. L. Callaghan, N. Tottenham, The stress acceleration hypothesis: Effects of early-life adversity on emotion circuits and behavior. *Curr. Opin. Behav. Sci.* **7**, 76–81 (2016).

50. K. L. Humphreys, M. C. Camacho, M. C. Roth, E. C. Estes, Prenatal stress exposure and multimodal assessment of amygdala–medial prefrontal cortex connectivity in infants. *Dev. Cogn. Neurosci.* **46**, 100877 (2020).

51. G. Schmidt Mellado, K. Pillay, E. Adams, A. Alarcon, F. Andritsou, M. M. Cobo, R. Evans Fry, S. Fitzgibbon, F. Moultrie, L. Baxter, R. Slater, The impact of premature extrauterine exposure on infants' stimulus-evoked brain activity across multiple sensory systems. *NeuroImage Clin.* **33**, 102914 (2022).

52. F. Lammertink, C. H. Vinkers, M. L. Tataranno, M. J. N. L. Benders, Premature birth and developmental programming: Mechanisms of resilience and vulnerability. *Front. Psychiatry* **11**, 531571 (2021).

53. J. L. Vincent, G. H. Patel, M. D. Fox, A. Z. Snyder, J. T. Baker, D. C. Van Essen, J. M. Zempel, L. H. Snyder, M. Corbetta, M. E. Raichle, Intrinsic functional architecture in the anaesthetized monkey brain. *Nature* **447**, 83–86 (2007).

54. K. H. Maier-Hein, P. F. Neher, J.-C. Houde, M.-A. Côté, E. Garyfallidis, J. Zhong, M. Chamberland, F.-C. Yeh, Y.-C. Lin, Q. Ji, W. E. Reddick, J. O. Glass, D. Q. Chen, Y. Feng, C. Gao, Y. Wu, J. Ma, H. Renjie, Q. Li, C.-F. Westin, S. Deslauriers-Gauthier, J. O. O. González, M. Paquette, S. St-Jean, G. Girard, F. Rheault, J. Sidhu, C. M. W. Tax, F. Guo, H. Y. Mesri, S. Dávid, M. Froeling, A. M. Heemskerk, A. Leemans, A. Boré, B. Pinsard, C. Bedetti, M. Desrosiers, S. Brambati, J. Doyon, A. Sarica, R. Vasta, A. Cerasa, A. Quattrone, J. Yeatman, A. R. Khan, W. Hodges, S. Alexander, D. Romascano, M. Barakovic, A. Auría, O. Esteban, A. Lemkaddem, J.-P. Thiran, H. E. Cetingul, B. L. Odry, B. Mailhe, M. S. Nadar, F. Pizzagalli, G. Prasad, J. E. Villalon-Reina, J. Galvis, P. M. Thompson, F. D. S. Requejo, P. L. Laguna, L. M. Lacerda, R. Barrett, F. Dell'Acqua, M. Catani, L. Petit, E. Caruyer, A. Daducci, T. B. Dyrby, T. Holland-Letz, C. C. Hilgetag, B. Stieltjes, M. Descoteaux, The challenge of mapping the human connectome based on diffusion tractography. *Nat. Commun.* **8**, 1349 (2017).

55. K. G. Schilling, V. Nath, C. Hansen, P. Parvathaneni, J. Blaber, Y. Gao, P. Neher, D. B. Aydogan, Y. Shi, M. Ocampo-Pineda, S. Schiavi, A. Daducci, G. Girard, M. Barakovic, J. Rafael-Patino, D. Romascano, G. Rensonnet, M. Pizzolato, A. Bates, E. Fischi, J.-P. Thiran, E. J. Canales-Rodríguez, C. Huang, H. Zhu, L. Zhong, R. Cabeen, A. W. Toga, F. Rheault, G. Theaud, J.-C. Houde, J. Sidhu, M. Chamberland, C.-F. Westin, T. B. Dyrby, R. Verma, Y. Rathi, M. O. Irfanoglu, C. Thomas, C. Pierpaoli, M. Descoteaux, A. W. Anderson, B. A. Landman, Limits to anatomical accuracy of diffusion tractography using modern approaches. *Neuroimage* **185**, 1–11 (2019).

56. M. Thiebaut de Schotten, F. Dell'Acqua, S. J. Forkel, A. Simmons, F. Vergani, D. G. M. M. Murphy, M. Catani, M. T. De Schotten, F. Dell'Acqua, S. J. Forkel, A. Simmons, F. Vergani, D. G. M. M. Murphy, M. Catani, A lateralized brain network for visuospatial attention. *Nat. Neurosci.* **14**, 1245–1246 (2011).

57. J. Dubois, M. Alison, S. J. Counsell, L. Hertz-Pannier, P. S. Hüppi, M. J. N. L. Benders, MRI of the neonatal brain: A review of methodological challenges and neuroscientific advances. *J. Magn. Reson. Imaging* **53**, 1318–1343 (2021).

58. D. C. Van Essen, S. Jbabdi, S. N. Sotiropoulos, C. Chen, K. Dikranian, T. Coalson, J. Harwell, T. E. J. Behrens, M. F. Glasser, H. Johansen-Berg. S. E. Behrens, Eds., Mapping connections in humans and non-human primates: aspirations and challenges for diffusion imaging, in *Diffusion MRI* (Academic Press, 2014), pp. 337–358.

59. D. Gwenaëlle, A. R. Groves, C. K. Tamnes, L. T. Westlye, E. P. Duff, A. Engvig, K. B. Walhovd, A. James, A. Gass, A. U. Monsch, P. M. Matthews, A. M. Fjell, S. M. Smith, H. Johansen-Berg, A common brain network links development, aging, and vulnerability to disease. *Proc. Natl. Acad. Sci. U.S.A.* **111**, 17648–17653 (2014).

60. L. Roumazeilles, N. Eichert, K. L. Bryant, D. Folloni, J. Sallet, S. Vijayakumar, S. Foxley, B. C. Tendler, S. Jbabdi, C. Reveley, L. Verhagen, L. B. Dershowitz, M. Guthrie, E. Flach, K. L. Miller, R. B. Mars, Longitudinal connections and the organization of the temporal cortex in macaques, great apes, and humans. *PLOS Biol.* **18**, e3000810 (2020).

61. K. L. Bryant, D. J. Ardesch, L. Roumazeilles, L. H. Scholtens, A. A. Khrapitchev, B. C. Tendler, W. Wu, K. L. Miller, J. Sallet, M. P. van den Heuvel, R. B. Mars, Diffusion MRI data, sulcal anatomy, and tractography for eight species from the Primate Brain Bank. *Brain Struct. Funct.* **226**, 2497–2509 (2021).

62. R. A. Benn, R. B. Mars, T. Xu, L. Rodríguez-esparragoza, P. Montesinos, G. Lopez-martin, J. Gonzalez-sanchez, E. P. Duff, B. Ibañez, A white matter atlas and common connectivity space facilitate the pig as a translational model in neuroscience. bioRxiv 2020.10.13.337436 [**Preprint**]. 14 October 2020. https://doi.org/10.1101/2020.10.13.337436.

63. C. Lebel, C. Beaulieu, Lateralization of the arcuate fasciculus from childhood to adulthood and its relation to cognitive abilities in children. *Hum. Brain Mapp.* **30**, 3563–3573 (2009).

64. P. L. Croxson, S. J. Forkel, L. Cerliani, M. Thiebaut de Schotten, Structural variability across the primate brain: A cross-species comparison. *Cereb. Cortex* **28**, 3829–3841 (2018).

65. D. Marie, M. Roth, R. Lacoste, B. Nazarian, A. Bertello, J.-L. Anton, W. D. Hopkins, K. Margiotoudi, S. A. Love, A. Meguerditchian, Left brain asymmetry of the planum temporale

in a nonhominid primate: Redefining the origin of brain specialization for language. *Cereb. Cortex* **28**, 1808–1815 (2018).

66. M. P. van den Heuvel, L. H. Scholtens, S. C. de Lange, R. Pijnenburg, W. Cahn, N. E. M. van Haren, I. E. Sommer, M. Bozzali, K. Koch, M. P. Boks, J. Repple, M. Pievani, L. Li, T. M. Preuss, J. K. Rilling, Evolutionary modifications in human brain connectivity associated with schizophrenia. *Brain* **142**, 3991–4002 (2019).

67. X. Liu, S. B. Eickhoff, S. Caspers, J. Wu, S. Genon, F. Hoffstaedter, R. B. Mars, I. E. Sommer, C. R. Eickhoff, J. Chen, R. Jardri, K. Reetz, I. Dogan, A. Aleman, L. Kogler, O. Gruber, J. Caspers, C. Mathys, K. R. Patil, Functional parcellation of human and macaque striatum reveals human-specific connectivity in the dorsal caudate. *Neuroimage* **235**, 118006 (2021).

68. A. Schuh, A. Makropoulos, E. Robinson, L. Cordero-Grande, E. Hughes, J. Hutter, A. Price, M. Murgasova, R. P. Teixeira, N. Tusor, J. Steinweg, S. Victor, M. Rutherford, J. Hajnal, A. D. Edwards, D. Rueckert, Unbiased construction of a temporally consistent morphological atlas of neonatal brain development. bioRxiv 251512 [**Preprint**]. 28 January 2018. https://doi.org/10.1101/251512.

69. M. Kuklisova-Murgasova, G. Quaghebeur, M. A. Rutherford, J. V. Hajnal, J. A. Schnabel, Reconstruction of fetal brain MRI with intensity matching and complete outlier removal. *Med. Image Anal.* **16**, 1550–1564 (2012).

70. J. L. R. Andersson, M. S. Graham, I. Drobnjak, H. Zhang, N. Filippini, M. Bastiani, Towards a comprehensive framework for movement and distortion correction of diffusion MR images: Within volume movement. *Neuroimage* **152**, 450–466 (2017).

71. M. Bastiani, J. L. R. Andersson, L. Cordero-Grande, M. Murgasova, J. Hutter, A. N. Price, A. Makropoulos, S. P. Fitzgibbon, E. Hughes, D. Rueckert, S. Victor, M. Rutherford, A. D. Edwards, S. M. Smith, J.-D. Tournier, J. V. Hajnal, S. Jbabdi, S. N. Sotiropoulos, Automated processing pipeline for neonatal diffusion MRI in the developing human connectome project. *Neuroimage* **185**, 750–763 (2019).

72. S. P. Fitzgibbon, S. J. Harrison, M. Jenkinson, L. Baxter, E. C. Robinson, M. Bastiani, J. Bozek, V. Karolis, L. Cordero Grande, A. N. Price, E. Hughes, A. Makropoulos, J. Passerat-Palmbach, A. Schuh, J. Gao, S.-R. Farahibozorg, J. O'Muircheartaigh, J. Ciarrusta, C. O'Keeffe, J. Brandon, T. Arichi, D. Rueckert, J V Hajnal, A. D. Edwards, S. M. Smith, E. Duff, J. Andersson, The developing Human Connectome Project (dHCP) automated resting-state functional processing framework for newborn infants. *Neuroimage* **223**, 117303 (2020).

73. D. C. Van Essen, S. M. Smith, D. M. Barch, T. E. Behrens, E. Yacoub, K. Ugurbil, The WU-Minn HCP Consortium: An overview, *Neuroimage* **80**, 62–79 (2013).

74. J. L. R. Andersson, M. Jenkinson, S. M. Smith, "Non-linear optimisation" (FMRIB Technical Report TR07JA1, FMRIB Centre, 2007).

75. D. C. Van Essen, Windows on the brain: The emerging role of atlases and databases in neuroscience. *Curr. Opin. Neurobiol.* **12**, 574–579 (2002).

76. S. Jbabdi, S. N. Sotiropoulos, A. M. Savio, M. Graña, T. E. J. Behrens, Model-based analysis of multishell diffusion MR data for tractography: How to get over fitting problems. *Magn. Reson. Med.* **68**, 1846–1855 (2012).

77. T. E. J. Behrens, H. J. Berg, S. Jbabdi, M. F. S. Rushworth, M. W. Woolrich, Probabilistic diffusion tractography with multiple fibre orientations: What can we gain? *Neuroimage* **34**, 144–155 (2007).

78. L. Baxter, F. Moultrie, S. Fitzgibbon, M. Aspbury, R. Mansfield, M. Bastiani, R. Rogers, S. Jbabdi, E. Duff, R. Slater, Functional and diffusion MRI reveal the neurophysiological basis of neonates' noxious-stimulus evoked brain activity. *Nat. Commun.* **12**, 2744 (2021).

79. A. Makropoulos, E. C. Robinson, A. Schuh, R. Wright, S. Fitzgibbon, J. Bozek, S. J. Counsell, J. Steinweg, K. Vecchiato, J. Passerat-Palmbach, G. Lenz, F. Mortari, T. Tenev, E. P. Duff, M. Bastiani, L. Cordero-Grande, E. Hughes, N. Tusor, J.-D. Tournier, J. Hutter, A. N. Price, R. P. A. G. Teixeira, M. Murgasova, S. Victor, C. Kelly, M. A. Rutherford, S. M. Smith, A. D. Edwards, J V Hajnal, M. Jenkinson, D. Rueckert, The developing human

connectome project: A minimal processing pipeline for neonatal cortical surface reconstruction. *Neuroimage* **173**, 88–112 (2018).

80. J. Bozek, A. Makropoulos, A. Schuh, S. Fitzgibbon, R. Wright, M. F. Glasser, T. S. Coalson, J. O'Muircheartaigh, J. Hutter, A. N. Price, L. Cordero-Grande, R. P. A. G. Teixeira, E. Hughes, N. Tusor, K. P. Baruteau, M. A. Rutherford, A. D. Edwards, J. V Hajnal, S. M. Smith, D. Rueckert, M. Jenkinson, E. C. Robinson, Construction of a neonatal cortical surface atlas using multimodal surface matching in the developing human connectome project. *Neuroimage* **179**, 11–29 (2018).

81. E. C. Robinson, K. Garcia, M. F. Glasser, Z. Chen, T. S. Coalson, A. Makropoulos, J. Bozek, R. Wright, A. Schuh, M. Webster, J. Hutter, A. Price, L. Cordero Grande, E. Hughes, N. Tusor, P. V. Bayly, D. C. Van Essen, S. M. Smith, A David Edwards, J. Hajnal, M. Jenkinson, B. Glocker, D. Rueckert, Multimodal surface matching with higher-order smoothness constraints. *Neuroimage* **167**, 453–465 (2018).

82. B. B. Avants, N. J. Tustison, G. Song, P. A. Cook, A. Klein, J. C. Gee, A reproducible evaluation of ANTs similarity metric performance in brain image registration. *Neuroimage* **54**, 2033–2044 (2011).

83. M. F. Glasser, S. N. Sotiropoulos, J. A. Wilson, T. S. Coalson, B. Fischl, J. L. Andersson, J. Xu, S. Jbabdi, M. Webster, J. R. Polimeni, D. C. Van Essen, M. Jenkinson; WU-Minn HCP Consortium, The minimal preprocessing pipelines for the human connectome project, *Neuroimage* **80**, 105–124 (2013).

84. M. Catani, F. Dell'Acqua, F. Vergani, F. Malik, H. Hodge, P. Roy, R. Valabregue, M. Thiebaut de Schotten, Short frontal lobe connections of the human brain. *Cortex* **48**, 273–291 (2012).

85. N. Makris, G. M. Papadimitriou, J. R. Kaiser, S. Sorg, D. N. Kennedy, D. N. Pandya, Delineation of the middle longitudinal fascicle in humans: A quantitative, in vivo, DT-MRI study. *Cereb. Cortex* **19**, 777–785 (2008).

86. J. Dubois, G. Dehaene-Lambertz, S. Kulikova, C. Poupon, P. S. Hüppi, L. Hertz-Pannier, The early development of brain white matter: A review of imaging studies in fetuses, newborns and infants. *Neuroscience* **276**, 48–71 (2014).

87. H. Takemura, F. Pestilli, K. S. Weiner, G. A. Keliris, S. M. Landi, J. Sliwa, F. Q. Ye, M. A. Barnett, D. A. Leopold, W. A. Freiwald, N. K. Logothetis, B. A. Wandell, Occipital white matter tracts in human and macaque. *Cereb. Cortex* **27**, 3346–3359 (2017).

88. S. Wakana, A. Caprihan, M. M. Panzenboeck, J. H. Fallon, M. Perry, R. L. Gollub, K. Hua, J. Zhang, H. Jiang, P. Dubey, A. Blitz, P. van Zijl, S. Mori, Reproducibility of quantitative tractography methods applied to cerebral white matter. *Neuroimage* **36**, 630–644 (2007).

89. M. Thiebaut de Schotten, F. Dell'Acqua, R. Valabregue, M. Catani, Monkey to human comparative anatomy of the frontal lobe association tracts. *Cortex* **48**, 82-96 (2012).

90. S. R. Heilbronner, S. N. Haber, Frontal cortical and subcortical projections provide a basis for segmenting the cingulum bundle: Implications for neuroimaging and psychiatric disorders. *J. Neurosci.* **34**, 10041–10054 (2014).

91. M. Catani, F. Dell'Acqua, M. Thiebaut de Schotten, A revised limbic system model for memory, emotion and behaviour. *Neurosci. Biobehav. Rev.* **37**, 1724–1737 (2013).

92. M. P. Harms, L. H. Somerville, B. M. Ances, J. Andersson, D. M. Barch, M. Bastiani, S. Y. Bookheimer, T. B. Brown, R. L. Buckner, G. C. Burgess, T. S. Coalson, M. A. Chappell, M. Dapretto, G. Douaud, B. Fischl, M. F. Glasser, D. N. Greve, C. Hodge, K. W. Jamison, S. Jbabdi, S. Kandala, X. Li, R. W. Mair, S. Mangia, D. Marcus, D. Mascali, S. Moeller, T. E. Nichols, E. C. Robinson, D. H. Salat, S. M. Smith, S. N. Sotiropoulos, M. Terpstra, K. M. Thomas, M. D. Tisdall, K. Ugurbil, A. van der Kouwe, R. P. Woods, L. Zöllei, D. C. Van Essen, E. Yacoub, Extending the human connectome project across ages: Imaging protocols for the lifespan development and aging projects. *Neuroimage* **183**, 972–984 (2018).
